# Insights from Impedance Spectroscopy in Perovskite Solar Cells with Self-Assembled Monolayers: Decoding SAM’s Tricks

**DOI:** 10.1021/acs.jpclett.4c03194

**Published:** 2025-02-24

**Authors:** Clara A. Aranda, Wenhui Li, Eugenia Martínez-Ferrero, Paul Pistor, Gerko Oskam, Emilio Palomares, Juan A. Anta

**Affiliations:** † Center for Nanoscience and Sustainable Technologies (CNATS), Department of Physical, Chemical and Natural Systems, 16772 Universidad Pablo de Olavide, 41013, Seville, Spain; ‡ 202569 Institute of Chemical Research of Catalonia (ICIQ), Avda. Paisos Catalans, 16, Tarragona, Spain; § Catalan Institution for Research and Advanced Studies (ICREA), 08010 Barcelona, Spain

## Abstract

Self-assembled monolayers (SAMs) are highly promising hole-transport layers for p-i-n perovskite solar cells, increasing photocurrent, reducing hysteresis and boosting photovoltage. However, the SAM’s exact role in maintaining those benefits remains elusive. This work demonstrates that SAMs enhance open-circuit voltage (*V*_oc_) and stability by suppressing surface recombination, as revealed by impedance spectroscopy. This is reflected in the time constants related to ionic dynamics, taking values from 10^–2^ to 10^–1^ s for PTAA-based samples and 10^–3^ s for SAM devices. X-ray photoelectron spectroscopy shows that SAMs chemically bind with hydroxyl groups on metal oxide substrates such as indium tin oxide, reducing ionic accumulation and preventing ion-induced *V*_oc_ losses. With minimal ionic dynamics, SAM-based devices achieve outstanding photovoltage and stability, confirming SAMs as pivotal in advancing perovskite cell performance.

## Graphical abstract

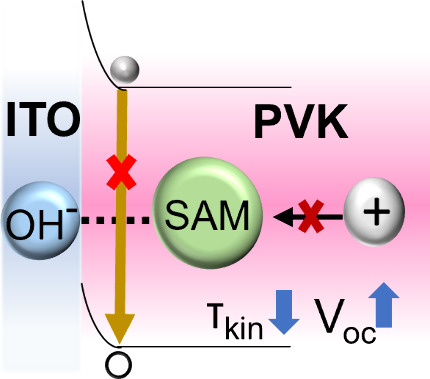

Perovskite-based solar cells (PSCs) represent a photovoltaic technology with great potential, but their instability under real-operation conditions still hinders their industrialization. This instability is caused, among other factors, by the migration of ions within the perovskite structure ABX_3_, (A: organic cation such as MA^+^, FA^+^; B: inorganic metal such as Pb or Sn; X: halogen, I, Br, Cl).^1,2^ This migration leads to inefficiencies in the devices, such as hysteresis in current–voltage (*jV*) measurements, positive and negative capacitance and open-circuit voltage (*V*_oc_) loss due to the additional recombination pathways introduced by the migrating ions.^3–5^

To reduce the harmful effects of ion dynamics, researchers focus on two main areas: (1) enhancing the intrinsic stability of the perovskite material itself and (2) improving the perovskite/contacts interfaces. Several strategies have been implemented for the bulk perovskite treatment. One of the most significant ones has been the introduction of alkali cations such as Cs and Rb. Cs stabilize the alpha phase of formamidinium, while Rb (I) acts as a blocking agent of diffusion pathways along the grain boundaries. This approach has led to the combinatorial design of perovskites, developing formulations such as Cs_*x*_(MA_0.17_FA_0.83_)_(100–*x*)_Pb­(I_0.83_Br_0.17_)_3_, with or without Rb, leading to a turning point in the perovskite field.^6,7^

However, while ions can have various effects on the perovskite bulk, their most pronounced impact is observed at the interfaces of the final device. This impact can vary significantly depending on the specific device architecture. PSCs can be broadly classified into two architectures based on the order of their layers: (i) regular configuration, that is n-i-p, and (ii) inverted configuration, known as p-i-n. In the n-i-p architecture, the perovskite layer is deposited on the n-type electron transport layer, ETL, followed by the p-type hole transport layer (HTL) and then the back metal contact, i.e., gold or silver. In n-i-p perovskite solar cells, commonly used electron transport layers include inorganic oxides (e.g., TiO_2_, SnO_2_, ZnO, Zn_2_SnO_2_, BaSnO_3_) and organic materials (PCBM, C_60_). Ideal ETLs should provide optimal band alignment, high transparency, and conductivity. Although TiO_2_ has been widely used, its photocatalytic activity and high processing temperatures limit scalability. SnO_2_ has emerged as a promising alternative due to its wide bandgap, high transparency, efficient charge transport, low-temperature processability, and stability, making it suitable for high-throughput PSC production. However, hysteresis and degradation remain challenges in SnO_2_-based PSCs that need to be addressed.^8^

The p-i-n architecture places the hole transport layer beneath the perovskite, enhancing stability and flexibility compared to n-i-p structures. This configuration requires an HTL that can endure the perovskite deposition process while supporting efficient perovskite growth. HTLs are primarily inorganic materials, like NiOx, or organic molecules. Inorganic HTLs offer excellent thermal stability and transparency but often require harsh processing conditions.^9^ Organic HTLs, on the other hand, can be solution-processed at lower temperatures and offer more flexibility in design. However, traditional organic HTLs, including polymers such as PEDOT:PSS (poly­(3,4-ethylenedioxythiophene):poly­(styrenesulfonate)) and PTAA (poly-[bis­(4-phenyl)­(2,4,6-trimethylphenyl)­amine]) that have been dominant in inverted PSCs, can be expensive or incompatible to the solvents used for the perovskite deposition.^10^ In this context, self-assembled monolayers have emerged as a promising alternative HTL for p-i-n PSCs. SAMs are ultrathin layers of organic molecules that can be chemically bonded to the underlying material. They offer several advantages: they are cheap, scalable, stable, easily processed and their properties can be easily tuned.^11,12^ Additionally, SAMs can modulate the energy levels at the interface between the selective contacts and the perovskite layer, which is crucial for efficient device operation. This modulation can be achieved through different anchoring groups attached to the SAMs: for example, phosphonic acid or carboxylic acid.

Tumen-Ulzii et al. have previously demonstrated that surface hydroxyl groups (OH^–^) present on SnO_2_ contribute to hysteresis and degradation of n-i-p PSCs by attracting positive ions to the interface.^13,14^ To suppress this accumulation of positive ions they chemically passivated SnO_2_ surface hydroxyl groups with a fullerene-based self-assembled monolayer.

A recent work has developed new carbazole-based self-assembled molecules as hole transport layers (HTLs) for p-i-n solar cells, achieving remarkable results. In these devices, SAMs are directly deposited on an ITO substrate, resulting in the following overall device architecture: Glass/ITO/SAM/Perovskite/C60-BCP/Cu. These cells demonstrated stable efficiencies exceeding 21%, with an impressive *V*_oc_ of 1.19 V for a perovskite bandgap of 1.63 eV.^15^ The solar cells maintained 80% of their initial conversion efficiency after 250 h of maximum power point tracking, representing notably improved results as compared with a reference sample using the traditional PTAA. These results have represented a significant achievement combining high efficiency and stability, essential for industrial application. However, the precise mechanism behind the high *V*_oc_ and stability remains unclear.

In this work, we explain these effects by analyzing impedance spectroscopy responses at the perovskite/contact interfaces in devices using either PTAA or SAM. The low-frequency region of the impedance spectra is generally associated with slow processes governed by the ionic motion within the perovskite devices.^16,17^ The analysis of the time constants corresponding to this region indicates a reduced ion dynamic by the presence of the SAM. The complete analysis of the impedance parameters reveals a decrease in the overall resistance in SAM-based devices, suggesting improved charge transport and reduced ion-induced recombination processes causing *V*_oc_ loss. Results that are corroborated with drift-diffusion simulations. X-ray photoelectron spectroscopy further confirms that SAMs chemically interact with bare ITO, lowering OH^–^ group concentrations that drive ionic migration.

The p-i-n perovskite solar devices with the architecture Glass/ITO/HTL/CsFAMA/C_60_/BCP/Cu, previously reported^15^ to exhibit improved *V*_oc_ and stability, have been evaluated. In these devices, the HTL was either PTAA, used as a reference, or SAM compounds (4-(3,6-bis­(2,4-dimethoxyphenyl)-9*H*-carbazol-9-yl)­benzoic acid, EADR03, see inset of Figure 1). The SAM compounds are based on a carbazole core functionalized with phenyl methoxy moieties. As described in ref 15, the enhancement in the device performance cannot be directly linked to the HOMO level of the SAM material, as PTAA has a deeper HOMO level. Instead, the improvement can be attributed to the effective charge transport and passivation effects of the SAM. However, the mechanism leading to this improvement needs to be elucidated.

**Figure 1:**
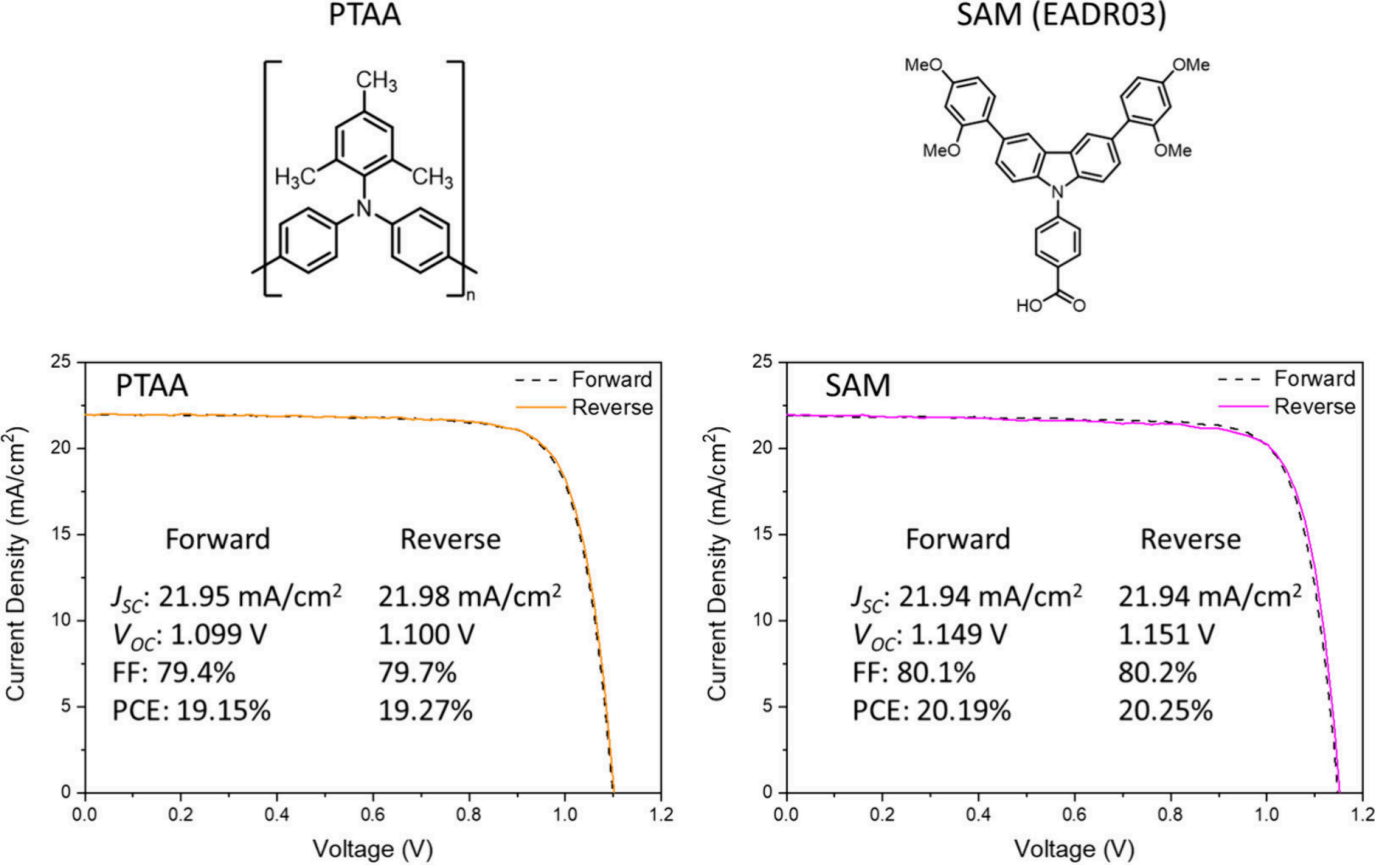
Current–voltage curves (measured at 100 mVs^–1^ scan rate) of the champion cells corresponding to the reference samples containing PTAA and the target samples using SAM as the HTL.

As mentioned in the introduction section, in an n-i-p perovskite device using SnO_2_ as ETL, hydroxyl groups (OH^–^) at the surface can act as defects. These defects lead to the localization of positive ions (MA^+^, FA^+^...) and halide vacancies from the perovskite bulk and their accumulation at the interface. Mobile ions cause hysteresis and surface recombination losses, leading to *V*_oc_ decrease and instability.^13^ By using SAM, −COO- groups can be formed through an esterification reaction with the OH^–^ groups from SnO_2_. This reaction deactivates the role of OH^–^ attracting positive perovskite ions to the surface, diminishing the harmful effects on the performance of the cells.

ITO is primarily composed of In_2_O_3_, doped with up to 20 wt % SnO_2_, and may feature terminal OH^–^ groups that can attract positively charged ions to its surface. If this hypothesis holds true, the ionic nature of this interaction should significantly influence the cyclic voltammetry (CV) response of the PTAA and SAM devices when measured at various scan rates. To investigate this, CV measurements were conducted at scan rates ranging from 10 to 500 mV/s for both types of devices. It is important to note that for the analysis of the electronic responses (CV and IS), the cells were transported to a different laboratory, exposing them to different conditions than those experienced by the champion cells shown in Figure 1.

In panels a and b of Figure 2, we show the corresponding behavior for PTAA-cells and SAM-cells, respectively. In both cases, an improvement in photocurrent proportional to the scan rate is observed. This effect has been previously attributed to a slow process involving the buildup of space charge near the contacts due to ionic displacement, which is associated with a capacitor-like discharge of current.^18,19^

**Figure 2:**
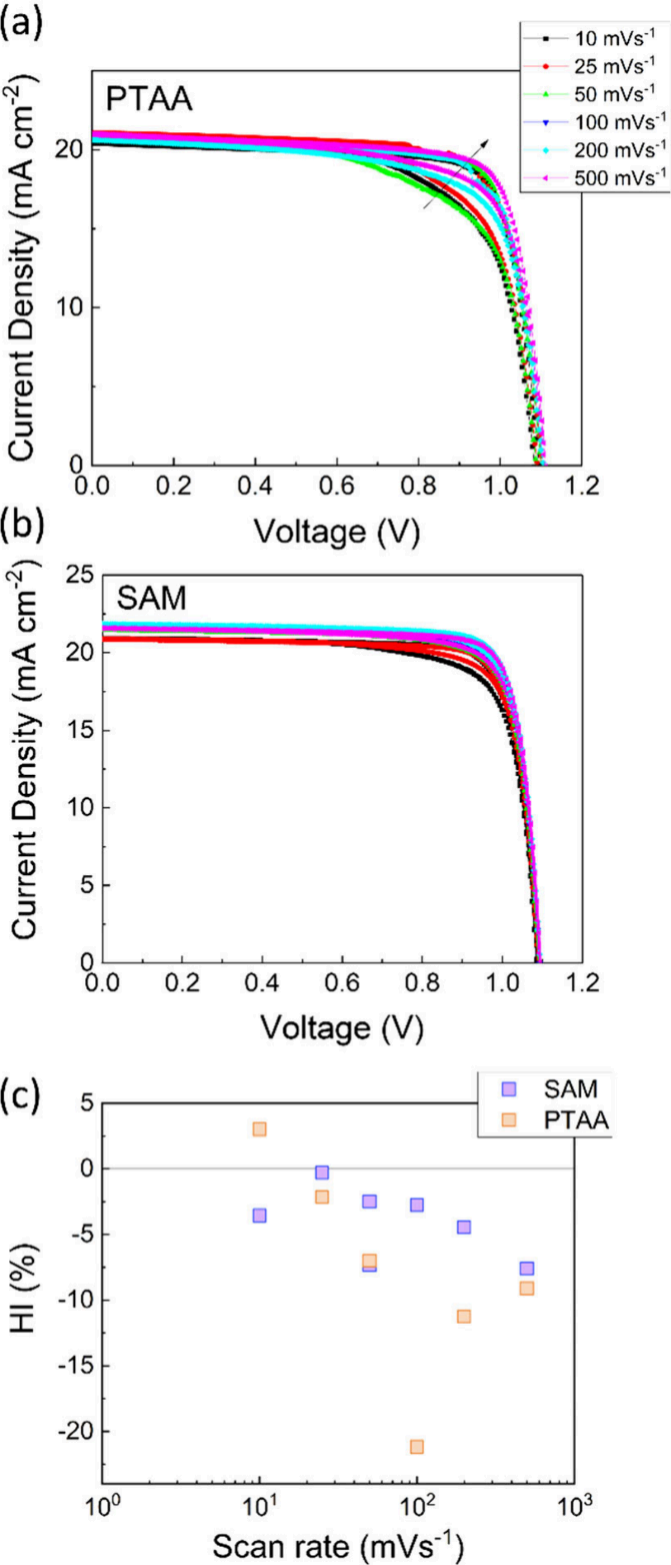
Current–voltage curves were measured at different scan rates for (a) the reference sample using PTAA as the HTL and (b) the device incorporating the SAM. (c) A comparison of the hysteresis index (calculated using eq 1) for both samples reveals a trend toward a negative hysteresis index (inverted hysteresis) in both cases, with this effect being more pronounced in the PTAA device. All measurements were performed under illumination from a white light LED (Golden DRAGON Plus, White LED, OSRAM Opto Semiconductors).

When analyzing the hysteresis index, defined as

1 $$HI=1-\frac{{PCE}_{FS}}{{PCE}_{RS}}$$

where PCE_FS_ represents the power conversion efficiency during the forward scan (short circuit to open circuit) and PCE_RS_ corresponds to the reverse scan (open circuit to short circuit), we observe a trend toward inverted hysteresis (negative values of hysteresis index) for both samples as the scan rate increases (Figure 2c).

Inverted hysteresis, where the efficiency obtained from the forward scan exceeds that of the reverse scan, is more pronounced in the case of the PTAA device. As discussed in our previous publication,^20^ significant trends toward inverted hysteresis serve as indicators of interfacial phenomena that may lead to undesirable recombination processes. The hysteresis data presented here reinforce the observation that the PTAA/perovskite interface is more affected by recombination mechanisms compared to the SAM-containing interface.

The evolution of the HI with the scan rate observed for both samples also aligns with the work reported by García-Rodríguez and co-workers.^21^ They combined experimental and modeling approaches to demonstrate the dependence of hysteresis on scan rate. The cell configuration (p-i-n, or n-i-p), the ion diffusion coefficient, and the nature of charge transport layers determine the crossover from normal to inverted hysteresis. A recent work reported by Clarke et al. analyzes these effects in depth, predicting the presence of inverted hysteresis by introducing a modified surface polarization model (m-SPM) that simplifies and helps to interpret the DD simulations.^22^ These observations reflect the distinct nature of the HTL materials in our study and highlights their influence on hysteresis behavior.

Impedance spectroscopy (IS) measures the electrical current response of a device under a steady-state voltage with a small AC perturbation across a range of frequencies (ω). The resulting impedance spectra provide valuable insights into the device’s behavior, which can be interpreted by fitting the data to an equivalent circuit model.^23^ In metal halide perovskite devices, the capacitance observed at low frequencies gives information about interfacial processes and is linked to the type of current–voltage hysteresis. Normal hysteresis is associated with positive capacitance, while inverted hysteresis has been correlated with negative capacitance.^5^ Negative capacitance appears as an *inductive behavior* in the impedance spectra, causing the imaginary part of the impedance -Im (Z) to drop below zero at low frequencies.

This connection between inverted hysteresis and negative capacitance arises from a common origin: ion-interface interactions causing additional recombination processes. These recombination mechanisms are associated with slow dynamics, such as the accumulation of ions or vacancies at the interface, which have been identified as a cause of open-circuit voltage (*V*_oc_) losses in perovskite solar cells.^24^ This phenomenon has been previously modeled by adding an R_L_-L branch to the equivalent circuit typically used to fit the impedance spectra. The time constant associated with this R_L_-L branch, denoted as τ_kin_, increases when the *inductive behavior* becomes more pronounced.^25,26^ The model used here associated with the equivalent circuit to fit the impedance response is related to the surface polarization model (SPM), widely reported and discussed in the literature. The SPM model was introduced to reduce the complexity of the DD analysis and facilitates the understanding of hysteresis. A more accurate version of this model had been recently developed by Clarke et al. This modified surface polarization model (m-SPM) was introduced to provide a rationale for the observation of inverted hysteresis, as is the case here. The m-SPM keeps the main approximation of the original SPM (that the concentration of ions is much larger than that of electronic carriers) but introduces the additional consideration that holes outnumber electrons due to the extraction barriers at the contacts. We note that this is also what we observe in the simulations we run () and that reproduce quite accurately the experiments.^22,27^

Said that, since SAM-based solar cells exhibit higher *V*_oc_, it is likely that recombination processes present in PTAA devices (which show lower *V*_oc_) are suppressed in SAM devices. This suppression is often attributed to effective surface passivation. Therefore, analyzing the impedance response of both sample types is crucial for understanding the mechanism through which the SAM enhances performance.

The focus here is to understand the recombination processes within the system, as they are directly linked to the observed improvements in open-circuit voltage (*V*_oc_) for the SAM samples. Given that *V*_oc_ is inherently tied to recombination, conducting impedance measurements near open-circuit conditions is essential.

However, when measurements are performed under illumination, especially across a wide frequency range needed to capture ionic contributions (including very low frequencies down to 10 mHz), the extended duration of such measurements can accelerate degradation in the samples. To ensure consistent and reliable conditions for both types of devices, we opted to conduct the impedance measurements in the dark while injecting carriers at 1.1 V, which closely approximates the *V*_oc_ conditions. These measurements provide similar insights into the recombination processes as those taken under illumination at *V*_oc_, but with the added benefit of minimizing degradation risks during the process.

Figures 3a show the Nyquist plots of the impedance spectra of both samples. In the impedance response of PSCs, the arcs corresponding to the high-frequency domain (from approximately 10^6^–10^2^ Hz) are commonly correlated with bulk processes, and the arcs corresponding to the intermedia-low-frequency region (from approximately 10^1^ to 10^–1^ Hz) have been linked to ionic and interfacial processes.^28,29^

**Figure 3:**
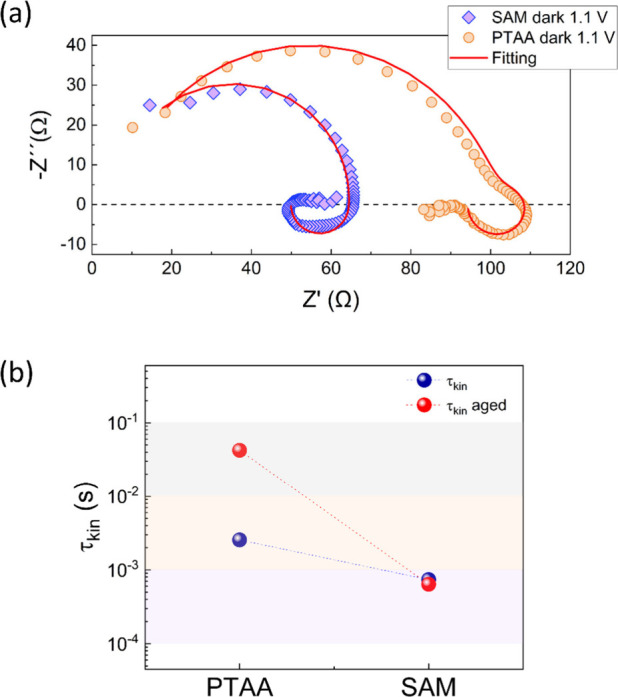
Impedance response showing the (a) Nyquist plot of PTAA (yellow color) and SAM (purple color) devices. (b) Time constants corresponding to both types of samples after the aging process (red) under humid room ambient. Cole–Cole plots and the corresponding fitting parameters of fresh and aged samples are shown in .

As we replace the PTAA (yellow) with SAM (purple), the high-frequency arc becomes smaller, indicating a reduction in the resistance. At the lowest frequencies, a decrease of -Im (Z) to values <0 appears for both cases, evidencing the signature of the negative capacitance or inductive behavior, which we modeled with a chemical inductor parameter characterized by a kinetic constant τ_kin_ (see for detailed explanation).^30^ This model has also been used to illustrate the electroforming process of perovskite-based memristors governed by ion dynamics, where a similar behavior can be observed. During this process, driven by the applied bias, a conductive pathway is formed, and the transition from a high resistance state (HRS) to a low resistance state (LRS) takes place, leading to a dramatic increase in the current. The electroforming is initiated by an accumulation of ions that gradually evolves to the stages determined by the faster processes of ion diffusion, as we recently reported (see for details).^31^

Considering this, we analyzed the impedance spectra of PTAA and SAM samples using the equivalent circuit that includes the inductive R_L_-L branch.

We would like to remark that the controversy presented in the field regarding the use of different models with different grades of accuracies (SPM, m-SPM, chemical inductor....) and, foremost, different equivalent circuits to interpret the physical processes governing perovskite devices, is well-known and much less negligible.^25,26,30,32^ This is the reason why many authors decide to focus just on the time constants directly extracted from the impedance spectra (Cole–Cole plot) without the need for a fitting. We would like to show here that the use of the *standard* SPM and its corresponding equivalent circuit associated, provides a trend of τ_kin_ values that fully agrees with the time constants directly extracted from the impedance plot, being well supported as well by the drift-diffusion simulations (DD) we have presented (available in the ), in agreement as well with previously reported works.^33^ In the following, we will discuss the values obtained both from the fitting using the EC and the direct extraction from the Cole–Cole plot of the impedance response.

Fitting results are presented in Table 1. In , panel (g) are presented the time constants extracted from the impedance plot.

**Table 1:** Extracted Parameters from IS of PTAA and SAM Samples under Dark Conditions and 1.1 V^a^

Sample	R_rec_ (kΩ)	R_c_ (kΩ)	R_L_ (kΩ)	L (H)	τ_kin_ (s)	τ_kin_ aged (s)
PTAA	0.10	6.9	0.46	1.2	0.0026	0.042
SAM	0.05	-	0.15	0.11	7.4 × 10^–4^	6.4 × 10^–4^

a See for the complete analysis.

In the high-frequency domain, replacing PTAA with SAM reduces overall resistance, as is evident from the shorter arc length. We propose that this reduced resistance in SAM devices, as seen in Figure 3a, suggests enhanced charge transport, similar to the electroforming process in memristors. However, this improvement should also manifest in the low-frequency domain, reflecting changes in ionic motion.

We proceed therefore to calculate the τ_kin_ through which the inductive behavior is defined (τ_kin_ = R_L_/L). The calculated values for both samples are shown in Figure 3b (blue dots) and are listed in Table 1. Here we observe a notable difference between the time constant τ_kin_ obtained for the PTAA device (2.6 × 10^–3^ s) and the SAM device (7.4 × 10^–4^ s), indicating that for PTAA a slower process is dominating the inductive behavior.^34,35^ The fact that this time is reduced in the SAM sample provides a piece of evidence of a lower ionic contribution, which, in turn, could favor the charge collection due to the lower overall recombination, causing the improvement in the *V*_oc_.

However, the SAM sample not only shows a better photovoltage performance but also an improved stability. To assess the long-term stability of the devices, we measured their impedance responses after aging under ambient conditions (variable temperature and humidity). The measurements were conducted under the same conditions as the previous experiments. Upon analyzing the spectra (see ) we obtained the results shown in Figure 3b (red dots). The τ_kin_ for the SAM sample remains nearly constant regardless of aging time and conditions. In contrast, the τ_kin_ for the PTAA sample increases by an order of magnitude after aging, indicating that PTAA’s lower stability is linked to a stronger ionic contribution, leading to slower processes with increased time constants. The Cole–Cole plots corresponding to both samples showing the evolution with aging are presented in Figure S4 in the .

To qualitatively support this observation, we performed drift-diffusion (DD) numerical simulations of the PTAA/perovskite/C60 structure, varying the ionic densities to replicate the effect of replacing PTAA with SAM and its effects on reducing the accumulation of ions at the interface. The simulations were conducted using SETFOS software (Fluxim Inc.) (details provided in the ).

As shown in , a decrease in ionic density (representing the situation of the SAM device) results in a shorter high-frequency (HF) arc, whereas higher ionic densities (representing the PTAA device) correspond to longer HF arcs, indicating higher resistances. Furthermore, the low-frequency (LF) region exhibits a much stronger inductive effect as the ionic density increases. In addition, corroborates larger concentrations of holes with respect to that of electrons, which can contribute to the inverted hysteresis as proposed in the m-SPM.

These results reinforce the hypothesis that SAM reduces the ionic contribution to the performance of perovskite devices. By limiting the presence of mobile ions, SAM improves interfacial stability and mitigates recombination processes, ultimately enhancing device performance. These results fully agree with the reported works by Thiesbrummel et al. and Lammar et al. where the DD calculations demonstrated that the accumulation of ions at the interface inhibits extraction, triggering recombination and producing performance losses.^36,37^

To investigate the proposed mechanism by which SAMs deactivate hydroxyl groups and reduce ionic migration, as suggested by our impedance results, we conducted a detailed XPS analysis. This analysis aimed to confirm any chemical changes at the interface by quantifying the relative abundance of OH^–^ groups. The study included three samples: the pristine ITO substrate, the ITO substrate with deposited PTAA, and the ITO substrate with deposited SAM. The findings, which provide insights into interfacial chemistry, are presented in Figure 4.

**Figure 4:**
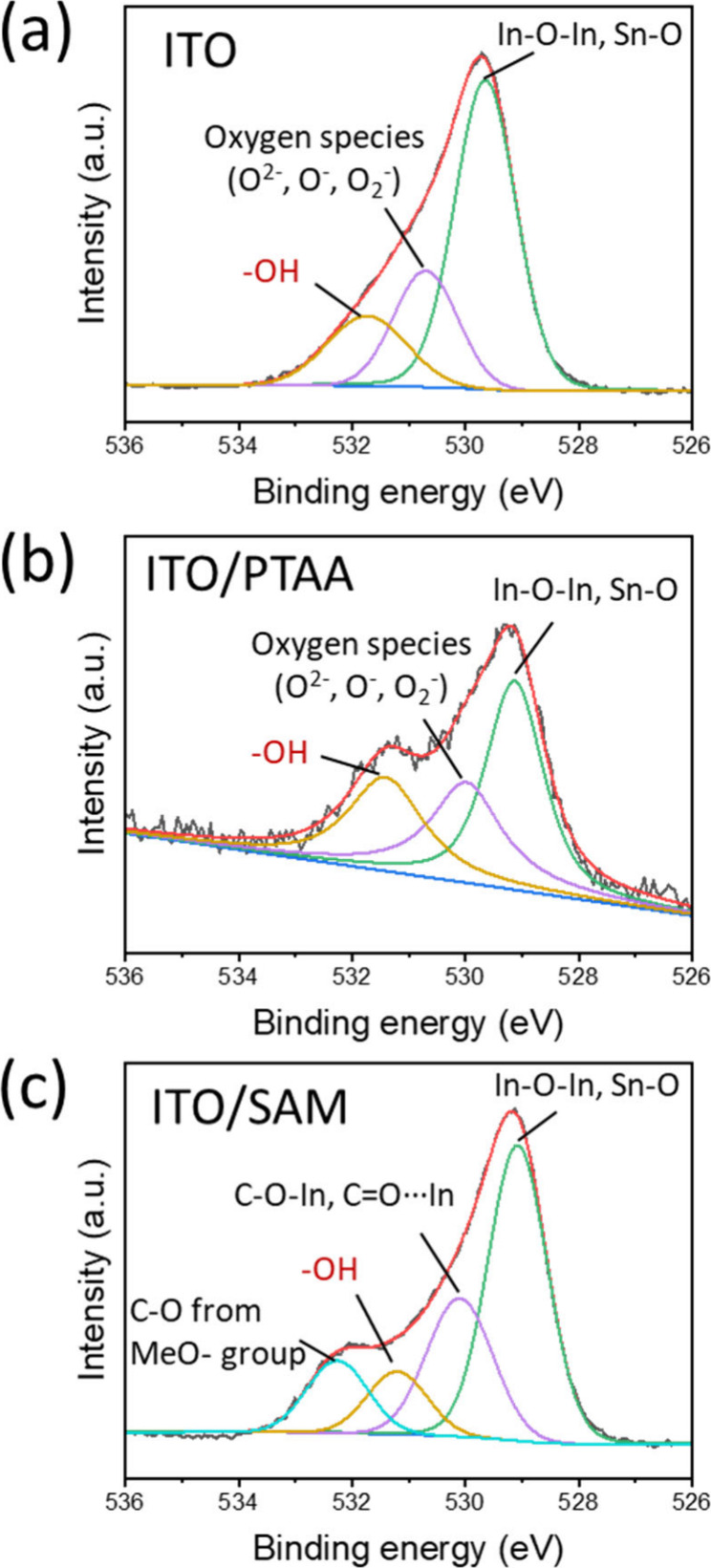
XPS analysis of O 1s region of ITO, ITO/PTAA and ITO/SAM substrates.

The XPS spectra of the ITO, PTAA, and SAM substrates are shown in Figures 4a, 4b, and 4c, respectively. Quantitative analysis of the relative abundance of OH^–^ groups reveals a decreasing trend from pristine ITO to PTAA to SAM, with SAM exhibiting the lowest OH^–^ content (see Table 2). This finding aligns perfectly with our initial hypothesis: SAM deposited directly onto ITO also forms −COO- groups via esterification between its carboxylic group and the OH^–^ groups from ITO, effectively deactivating them similarly to SnO_2_-based n-i-p structures. This observation is also in accord with the impedance response analysis outlined before. Deactivating OH^–^ groups reduces ionic contributions, decreasing time constants and diminishing ionic influence in the performance of the sample. This reduction benefits charge transport and extraction, as overall resistance decreases with reduced ionic motion.

**Table 2:** Quantitative Analysis of the −OH Proportion Analyzing the O 1s Spectra^a^

**Sample**	**–OH proportion**
ITO	17%
ITO/PTAA	27%
ITO/SAM	11%

a This analysis was made similarly to the previously reported in ref 15.

Similar results for n-i-p perovskite devices were recently reported by our group, showing that doping TiO_2_ with alkali metals significantly reduced ionic contributions, achieving a stable *V*_oc_ of 1.65 V for a 2.3 eV bandgap perovskite material.^38^ Blocking ionic migration minimized ion accumulation at the interface, enhancing *V*_oc_ and stability even under ambient conditions without encapsulation. Impedance measurements confirmed reduced surface recombination and the absence of inductive behavior. XPS analysis further revealed that a chemical interaction prevented cation accumulation at the interface, thereby reducing surface recombination, similar to our current case.

Our proposed mechanism is shown in Figure 5: In PTAA-based devices, perovskite ions tend to accumulate at the interface, promoting surface recombination mechanisms (in this case the perovskite/HTL interface) extensively reported elsewhere.^39,40^ This surface recombination leads to a decrease in the electronic charge density, leading to a reduced *V*_oc_ and poor charge extraction. In the case of SAM, the interaction between the carboxylic anchoring group and the free OH^–^ present at the ITO surface impedes the electrostatic interaction between the OH^–^ and the positively charged ions/vacancies from the perovskite. Consequently, the *deactivation* of the OH^–^ moieties prevents the accumulation of positive species at the interface, reducing hysteresis, favoring charge separation and diminishing the surface recombination, leading to a higher electronic charge density, which explains the enhancement of the *V*_oc_ typically observed in the literature when SAM is used in perovskite solar cells. The drift-diffusion numerical simulations (see ) confirm this interpretation. In addition to the deactivation of OH^–^ by SAM, the perovskite crystal morphology and crystallinity influenced by PTAA and SAM may also play a significant role in suppressing ion accumulation at the interface. This phenomenon has been explored in our previous studies.^41^

**Figure 5:**
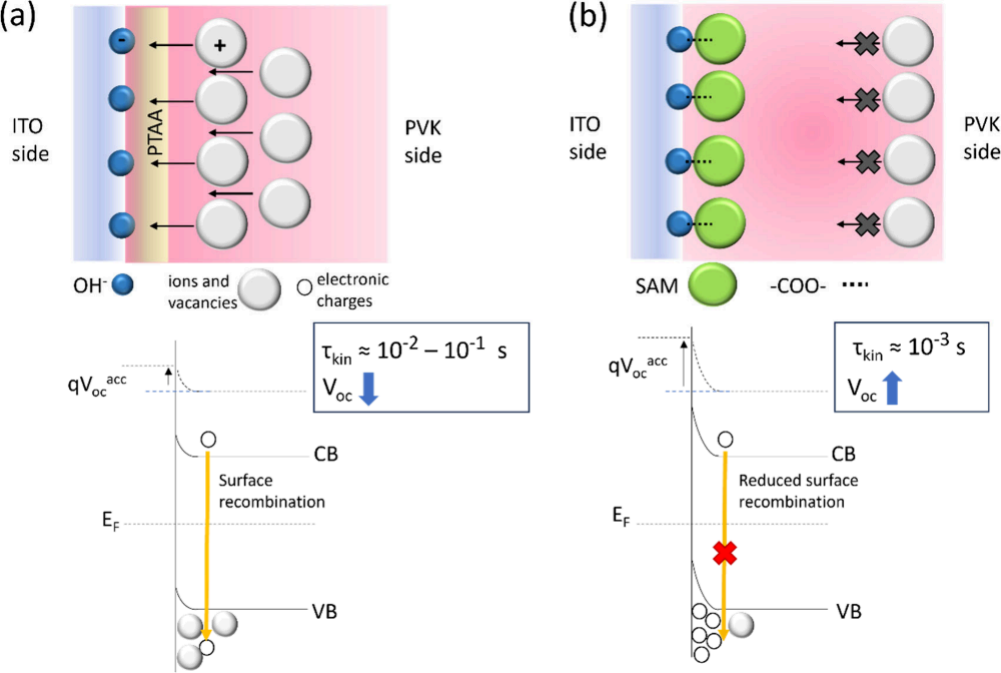
Scheme of the mechanism proposed explaining the chemical *trick* that SAMs play at the interface between perovskite and ITO. From top left to bottom right: Top left: Situation occurring for PTAA-based devices, with ^–^OH groups at the ITO surface, where positively charged species accumulate at the interface. Top right: Situation with SAM, where this ionic accumulation is blocked through the formation of −COO- groups. Bottom left: Band bending scenario where the ionic accumulation leads to positive charges at the interface, effectively reducing the band bending toward the hole selective contact. This, in turn, leads to a reduced built-in voltage and slower charge extraction across the interface. Surface recombination is present and the *V*_oc_ is reduced (*V*_oc_^*acc*^ represents the voltage due to the accumulation of charges at the interface). Bottom right: When the SAM is present, ionic accumulation is reduced and the measured time constant is much smaller. The band bending toward the selective hole contact increases and surface recombination is drastically reduced, which provides higher *V*_oc_.

In summary, we have explained how self-assembled molecules in p-i-n PSCs architectures can provide outstanding stability together with an improved open circuit voltage for a 1.63 eV bandgap perovskite material. We have performed a comprehensive impedance spectroscopy analysis of devices containing SAMs, using PTAA as reference material. A combination of IS experiments and numerical modeling has identified a notable decrease in the ionic contribution in the performance of the SAM-based perovskite devices. The surface recombination, responsible for *V*_oc_ losses, is diminished by a chemical interaction, confirmed by XPS analysis, between the OH^–^ groups present in the ITO substrate and the carboxylic groups of the SAM. This interaction avoids the accumulation of the positively charged ions/vacancies at the interface. This is revealed by the analysis of the low-frequency impedance response, where the time constants associated, τ_kin_, take values of ≈10^–3^ s for the SAM device and ≈10^–2^ – 10^–1^ s in the PTAA sample, the latest associated with ionic accumulation. There is no accumulation of ions/vacancies at the interface in the case of SAM, which reduces the associated surface recombination mechanism and leads to higher *V*_oc_ and better charge extraction capability. This effect is expected to be observed in other SAMs that chemically bond to the ITO surface. Therefore, this manuscript just provides more fundamental knowledge to understand the improvement seen on SAM-based devices.

## Supplementary Material





## Notes

Additional impedance plots, further analyzed impedance parameters, additional explanations and drift-diffusion simulations (PDF)
Transparent Peer Review report available (PDF)

## References

[1] Tenuta E., Zheng C., Rubel O. (2016). Thermodynamic Origin of Instability
in Hybrid Halide Perovskites. Sci. Rep.

[2] Thiesbrummel J., Shah S., Gutierrez-Partida E., Zu F., Peña-Camargo F., Zeiske S., Diekmann J., Ye F., Peters K. P., Brinkmann K. O., Caprioglio P., Dasgupta A., Seo S., Adeleye F. A., Warby J., Jeangros Q., Lang F., Zhang S., Albrecht S., Riedl T., Armin A., Neher D., Koch N., Wu Y., Le Corre V. M., Snaith H., Stolterfoht M. (2024). Ion-Induced
Field Screening as a
Dominant Factor in Perovskite Solar Cell Operational Stability. Nat. Energy.

[3] Richardson G., O’Kane S. E. J., Niemann R. G., Peltola T. A., Foster J. M., Cameron P. J., Walker A. B. (2016). Can Slow-Moving
Ions Explain Hysteresis
in the Current–Voltage Curves of Perovskite Solar Cells?. Energy Environ. Sci..

[4] Kirchartz T., Krückemeier L., Unger E. L. (2018). Research Update: Recombination and
Open-Circuit Voltage in Lead-Halide Perovskites. APL Mater..

[5] Alvarez A. O., Arcas R., Aranda C. A., Bethencourt L., Mas-Marzá E., Saliba M., Fabregat-Santiago F. (2020). Negative Capacitance
and Inverted Hysteresis: Matching Features in Perovskite Solar Cells. J. Phys. Chem. Lett..

[6] Saliba M., Matsui T., Seo J.-Y., Domanski K., Correa-Baena J.-P., Nazeeruddin M. K., Zakeeruddin S. M., Tress W., Abate A., Hagfeldt A., Grätzel M. (2016). Cesium-Containing Triple Cation Perovskite
Solar Cells: Improved Stability, Reproducibility and High Efficiency. Energy Environ. Sci..

[7] Saliba M., Matsui T., Domanski K., Seo J. Y., Ummadisingu A., Zakeeruddin S. M., Correa-Baena J. P., Tress W. R., Abate A., Hagfeldt A., Grätzel M. (2016). Incorporation of Rubidium Cations
into Perovskite Solar Cells Improves Photovoltaic Performance. Science (1979).

[8] Bu T., Li J., Zheng F., Chen W., Wen X., Ku Z., Peng Y., Zhong J., Cheng Y.-B., Huang F. (2018). Universal
Passivation Strategy to Slot-Die Printed SnO2 for Hysteresis-Free
Efficient Flexible Perovskite Solar Module. Nat. Commun..

[9] Sajid S., Alzahmi S., Salem I. Ben, Park J., Obaidat I. M. (2023). Inorganic
Hole Transport Materials in Perovskite Solar Cells Are Catching Up. Mater. Today Energy.

[10] Zhang C., Wei K., Hu J., Cai X., Du G., Deng J., Luo Z., Zhang X., Wang Y., Yang L., Zhang J. (2023). A Review on
Organic Hole Transport Materials for Perovskite Solar Cells: Structure,
Composition and Reliability. Mater. Today.

[11] Li W., Martínez-Ferrero E., Palomares E. (2024). Self-Assembled
Molecules as Selective Contacts for Efficient and Stable Perovskite
Solar Cells. Mater. Chem. Front.

[12] Puerto
Galvis C. E., González Ruiz D. A., Martínez-Ferrero E., Palomares E. (2024). Challenges in the Design and Synthesis of Self-Assembling
Molecules as Selective Contacts in Perovskite Solar Cells. Chem. Sci..

[13] Tumen-Ulzii G., Matsushima T., Klotz D., Leyden M. R., Wang P., Qin C., Lee J.-W., Lee S.-J., Yang Y., Adachi C. (2020). Hysteresis-Less
and Stable Perovskite Solar Cells with a Self-Assembled Monolayer. Commun. Mater..

[14] Jeon Y.-S., Kang D.-H., Kim J.-H., Park N.-G. (2023). Stability
and Efficiency
Improvement of Perovskite Solar Cells by Surface Hydroxyl Defect Passivation
of SnO2 Layer with 4-Fluorothiophenol. J. Mater.
Chem. A Mater..

[15] Aktas E., Phung N., Köbler H., González D. A., Méndez M., Kafedjiska I., Turren-Cruz S.-H., Wenisch R., Lauermann I., Abate A., Palomares E. (2021). Understanding
the Perovskite/Self-Assembled Selective Contact Interface for Ultra-Stable
and Highly Efficient p–i–n Perovskite Solar Cells. Energy Environ. Sci..

[16] Fabregat-Santiago F., Garcia-Belmonte G., Mora-Seró I., Bisquert J. (2011). Characterization of
Nanostructured Hybrid and Organic Solar Cells by Impedance Spectroscopy. Phys. Chem. Chem. Phys..

[17] von
Hauff E. (2019). Impedance Spectroscopy for Emerging Photovoltaics. J. Phys. Chem. C.

[18] Bisquert, J. ; Garcia-Belmonte, G. ; Mora-Sero, I. Characterization of Capacitance, Transport and Recombination Parameters in Hybrid Perovskite and Organic Solar Cells. In Unconventional Thin Film Photovoltaics: Organic and Perovskite Solar Cells; Da Como, E. , De Angelis, F. , Snaith, H. , Walker, A. , Eds.; The Royal Society of Chemistry, 2016, ch. 3, pp. 57-106.

[19] Jacobs D. A., Shen H., Pfeffer F., Peng J., White T. P., Beck F. J., Catchpole K. R. (2018). The Two Faces of Capacitance: New
Interpretations for Electrical Impedance Measurements of Perovskite
Solar Cells and Their Relation to Hysteresis. J. Appl. Phys..

[20] Nemnes G. A., Besleaga C., Stancu V., Dogaru D. E., Leonat L. N., Pintilie L., Torfason K., Ilkov M., Manolescu A., Pintilie I. (2017). Normal and Inverted Hysteresis in Perovskite Solar
Cells. J. Phys. Chem. C.

[21] García-Rodríguez R., Riquelme A. J., Cowley M., Valadez-Villalobos K., Oskam G., Bennett L. J., Wolf M. J., Contreras-Bernal L., Cameron P. J., Walker A. B., Anta J. A. (2022). Inverted Hysteresis
in n–i–p and p–i–n Perovskite Solar Cells. Energy Technology.

[22] Clarke W., Cowley M. V., Wolf M. J., Cameron P., Walker A., Richardson G. (2023). Inverted Hysteresis
as a Diagnostic Tool for Perovskite
Solar Cells: Insights from the Drift-Diffusion Model. J. Appl. Phys..

[23] Guerrero A., Bisquert J., Garcia-Belmonte G. (2021). Impedance
Spectroscopy of Metal Halide
Perovskite Solar Cells from the Perspective of Equivalent Circuits. Chem. Rev..

[24] Aranda C., Guerrero A., Bisquert J. (2019). Ionic Effect
Enhances Light Emission
and the Photovoltage of Methylammonium Lead Bromide Perovskite Solar
Cells by Reduced Surface Recombination. ACS
Energy Lett..

[25] Ravishankar S., Almora O., Echeverría-Arrondo C., Ghahremanirad E., Aranda C., Guerrero A., Fabregat-Santiago F., Zaban A., Garcia-Belmonte G., Bisquert J. (2017). Surface Polarization
Model for the Dynamic Hysteresis of Perovskite Solar Cells. J. Phys. Chem. Lett..

[26] Ghahremanirad E., Bou A., Olyaee S., Bisquert J. (2017). Inductive Loop in the Impedance Response
of Perovskite Solar Cells Explained by Surface Polarization Model. J. Phys. Chem. Lett..

[27] Clarke W., Richardson G., Cameron P. (2024). Understanding the Full Zoo of Perovskite
Solar Cell Impedance Spectra with the Standard Drift-Diffusion Model. Adv. Energy Mater..

[28] Garcia-Belmonte G., Guerrero A., Bisquert J. (2013). Elucidating
Operating Modes of Bulk-Heterojunction
Solar Cells from Impedance Spectroscopy Analysis. J. Phys. Chem. Lett..

[29] Bisquert J., Bertoluzzi L., Mora-Sero I., Garcia-Belmonte G. (2014). Theory of
Impedance and Capacitance Spectroscopy of Solar Cells with Dielectric
Relaxation, Drift-Diffusion Transport, and Recombination. J. Phys. Chem. C.

[30] Bisquert J., Guerrero A. (2022). Chemical Inductor. J. Am. Chem.
Soc..

[31] Fernandez-Guillen I., Aranda C. A., Betancur P. F., Vallés-Pelarda M., Momblona C., Ripolles T. S., Abargues R., Boix P. P. (2024). Perovskite
Thin Single Crystal for a High Performance and Long Endurance Memristor. Adv. Electron Mater..

[32] Moia D., Gelmetti I., Calado P., Fisher W., Stringer M., Game O., Hu Y., Docampo P., Lidzey D., Palomares E., Nelson J., Barnes P. R. F. (2019). Ionic-to-Electronic
Current Amplification in Hybrid Perovskite Solar Cells: Ionically
Gated Transistor-Interface Circuit Model Explains Hysteresis and Impedance
of Mixed Conducting Devices. Energy Environ.
Sci..

[33] Riquelme A., Bennett L. J., Courtier N. E., Wolf M. J., Contreras-Bernal L., Walker A. B., Richardson G., Anta J. A. (2020). Identification of
Recombination Losses and Charge Collection Efficiency in a Perovskite
Solar Cell by Comparing Impedance Response to a Drift-Diffusion Model. Nanoscale.

[34] Wang H., Guerrero A., Bou A., Al-Mayouf A. M., Bisquert J. (2019). Kinetic and Material Properties of
Interfaces Governing
Slow Response and Long Timescale Phenomena in Perovskite Solar Cells. Energy Environ. Sci..

[35] Seijas-Bellido J. A., Samanta B., Valadez-Villalobos K., Gallardo J. J., Navas J., Balestra S. R. G., Madero
Castro R. M., Vicent-Luna J. M., Tao S., Toroker M. C., Anta J. A. (2022). Transferable Classical Force Field
for Pure and Mixed Metal Halide Perovskites Parameterized from First-Principles. J. Chem. Inf Model.

[36] Thiesbrummel J., Le Corre V. M., Peña-Camargo F., Perdigón-Toro L., Lang F., Yang F., Grischek M., Gutierrez-Partida E., Warby J., Farrar M. D., Mahesh S., Caprioglio P., Albrecht S., Neher D., Snaith H. J., Stolterfoht M. (2021). Universal
Current Losses in Perovskite Solar Cells Due to Mobile Ions. Adv. Energy Mater..

[37] Lammar S., Escalante R., Riquelme A. J., Jenatsch S., Ruhstaller B., Oskam G., Aernouts T., Anta J. A. (2022). Impact of Non-Stoichiometry
on Ion Migration and Photovoltaic Performance of Formamidinium-Based
Perovskite Solar Cells. J. Mater. Chem. A Mater..

[38] Aranda C. A., Alvarez A. O., Chivrony V. S., Das C., Rai M., Saliba M. (2024). Overcoming Ionic Migration in Perovskite
Solar Cells
through Alkali Metals. Joule.

[39] Zarazua I., Han G., Boix P. P., Mhaisalkar S., Fabregat-Santiago F., Mora-Seró I., Bisquert J., Garcia-Belmonte G. (2016). Surface Recombination
and Collection Efficiency in Perovskite Solar Cells from Impedance
Analysis. J. Phys. Chem. Lett..

[40] Zarazua I., Bisquert J., Garcia-Belmonte G. (2016). Light-Induced Space-Charge Accumulation
Zone as Photovoltaic Mechanism in Perovskite Solar Cells. J. Phys. Chem. Lett..

[41] Li W., Cariello M., Méndez M., Cooke G., Palomares E. (2023). Self-Assembled
Molecules for Hole-Selective Electrodes in Highly Stable and Efficient
Inverted Perovskite Solar Cells with Ultralow Energy Loss. ACS Appl. Energy Mater..

