# Supplementary material for: Insights from Impedance Spectroscopy in Perovskite Solar Cells with Self-Assembled Monolayers: Decoding SAM’s Tricks
Source: J Phys Chem Lett. 2025 Feb 24;16(9):2301–8. doi: 10.1021/acs.jpclett.4c03194 (PMC12142668; doi:10.1021/acs.jpclett.4c03194)
Supplement: Supplementary file 1 [file jz4c03194_si_001.pdf]

Supporting Information

Insights from Impedance Spectroscopy in Perovskite Solar Cells with Self-Assembled Monolayers: Decoding SAM´s Tricks

Clara A. Aranda[1]*, Wenhui Li[2], Eugenia Martínez-Ferrero[2], Paul Pistor[1], Gerko Oskam[1], Emilio Palomares[2,3], Juan Anta[1]*

[1]Center for Nanoscience and Sustainable Technologies (CNATS), Department of Physical, Chemical and Natural Systems, Universidad Pablo de Olavide, 41013, Seville, Spain.
[2]Institute of Chemical Research of Catalonia (ICIQ), Avda. Paisos Catalans, 16, Tarragona, Spain.
[3] Catalan Institution for Research and Advanced Studies (ICREA), 08010 Barcelona, Spain.

Email: cparaalo@upo.es

20 February 2025

<u>Impedance parameters of devices before the aging</u>

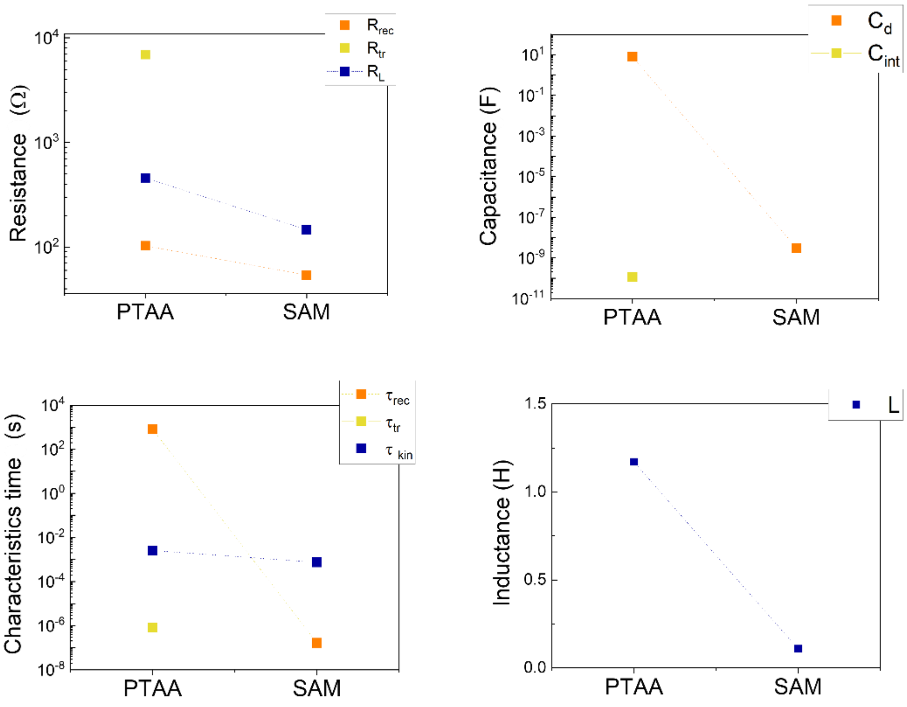

Figure S1. Fitting parameters of the impedance spectra of both samples. From left to right and top to bottom: resistance, capacitance, characteristic time, and inductance.

Impedance parameters of devices after the aging

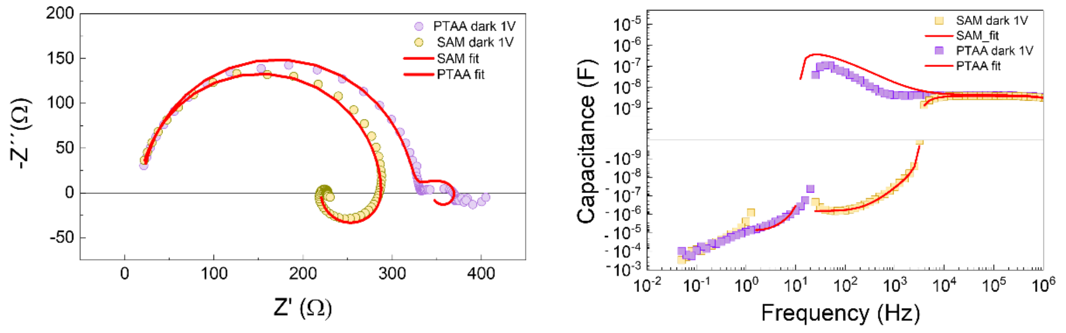

Figure S2. Nyquist plot (a) and capacitance versus frequency response (b) of aged samples under room conditions. Note the more pronounced NC in the PTAA-based device (purple color). Also, comparing with the simulations of Figure S1, it can be observed that for both theoretical and experimental cases, when the ionic density is very high, a second arc starts to appear in the HF domain.

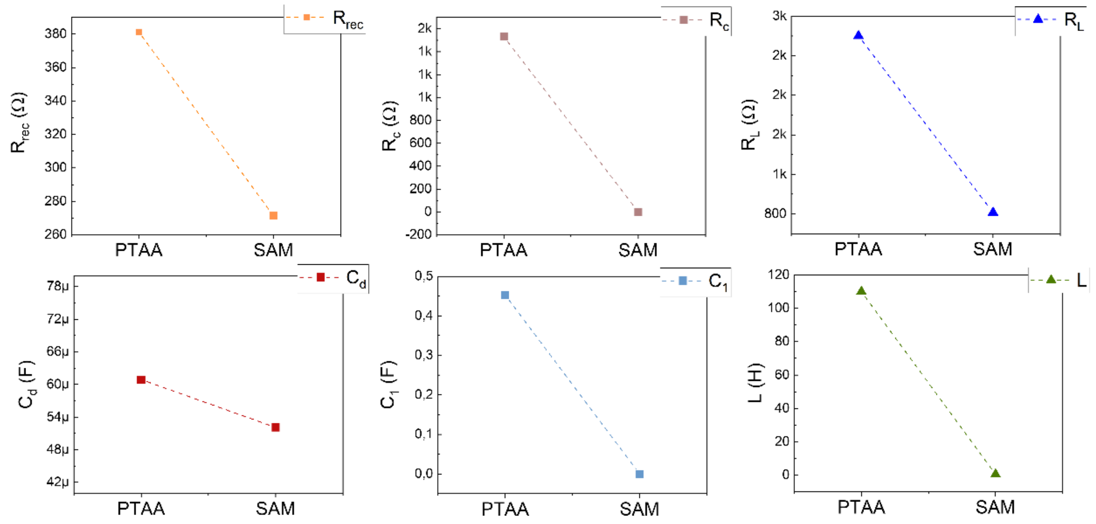

Figure S3. Fitting parameters of the aged devices.

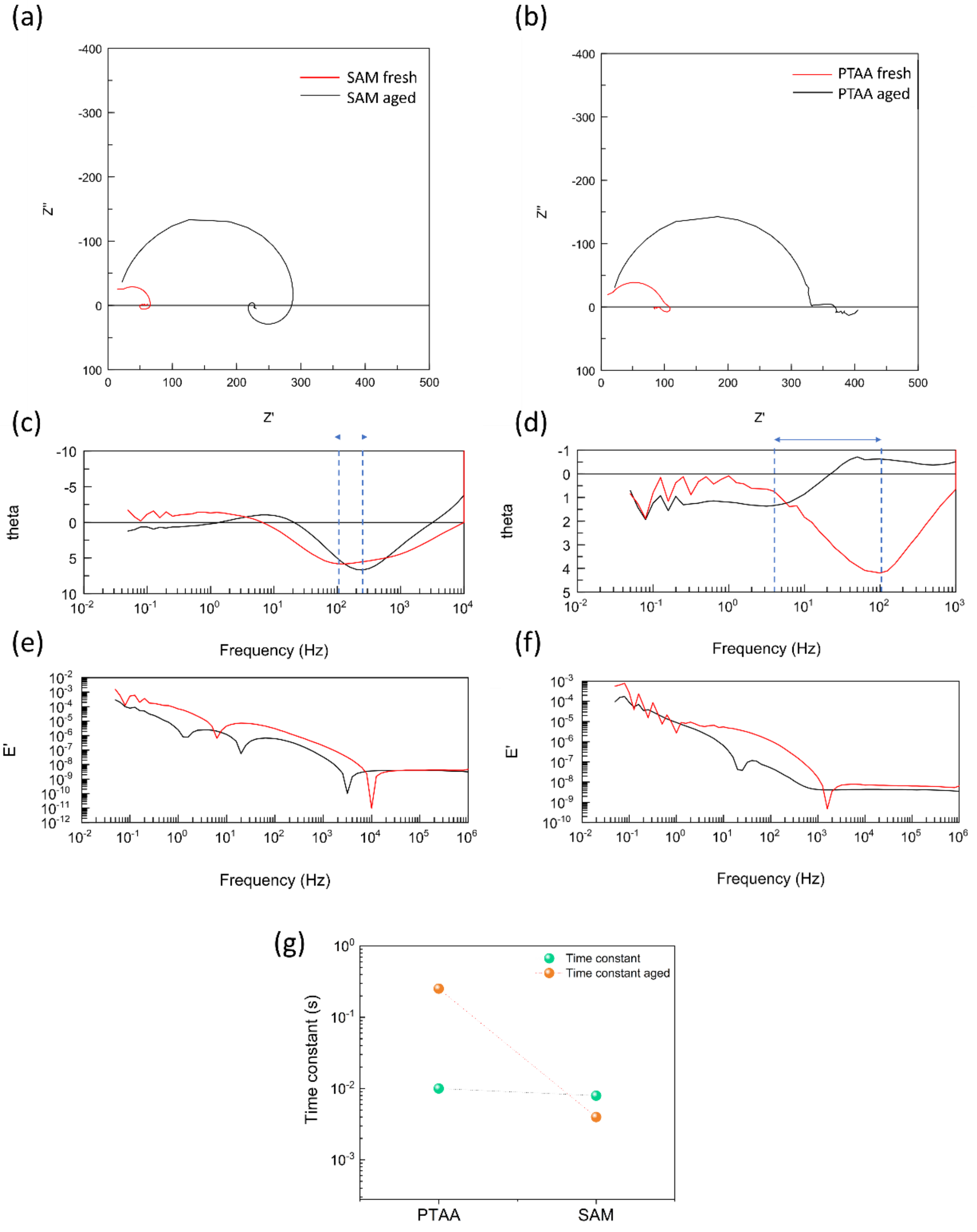

Figure S4. Comparison of the impedance responses of fresh and aged devices. Panels (a), (b), and (c) correspond to the Nyquist plot, phase, and capacitance of SAM devices, respectively. Panels (d), (e), and (f) represent the same parameters for PTAA devices. Color code: red for fresh devices, black for aged devices. Notably, for PTAA samples, aging results in a significant shift of the time constant to much slower frequencies (e), in contrast to SAM devices, where the time constant remains nearly unchanged (b). This is clearly seen in the panel (g) showing the time constants extracted directly from the Cole-Cole plot. The green color represents the values for the fresh devices, whereas the orange color corresponds to the ones after aging. Note that this trend aligns perfectly with the trend obtained from the fitting of the EC used as well as with the DD simulations.

Table S1: Frequencies and time constants extracted from the Cole-Cole plot.

Sample	*Frequency (Hz)*	*Frequency aged (Hz)*	*τ (s)*	*τ aged (s)*
**PTAA**	99.87	3.97	0.010	0.252
**SAM**	125.73	250.9	7.95×10^{-3}	3.98×10^{-3}

Drift-diffusion simulations using *Setfos* software from *Fluxim*

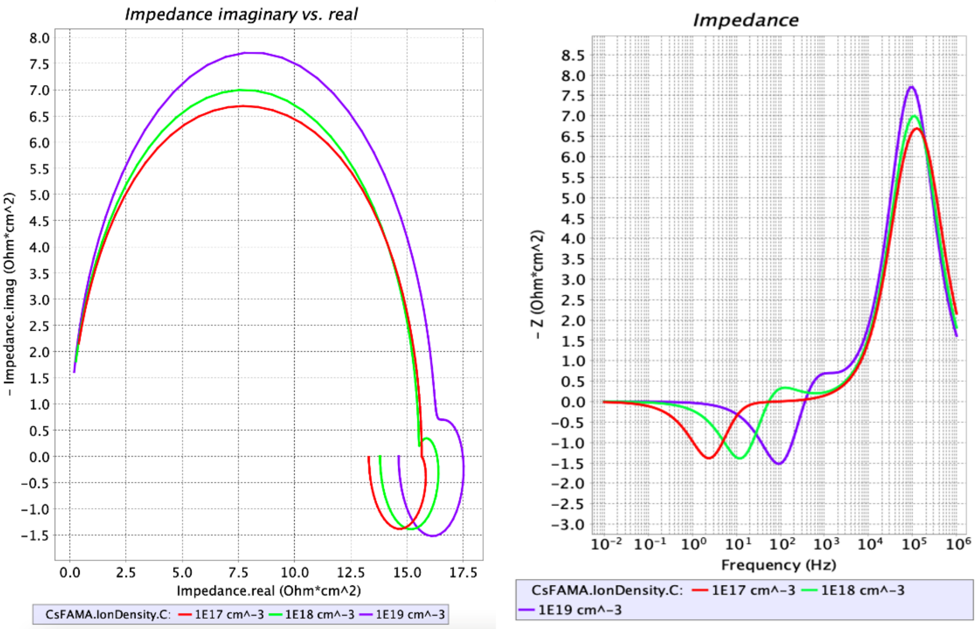

Figure S5. From left to right: Nyquist plot showing the increase in the HF arc length with the increase of the ionic density. On the right side, the LF performance is shown, where the increase in the ionic density gives a much more pronounced inductive effect.

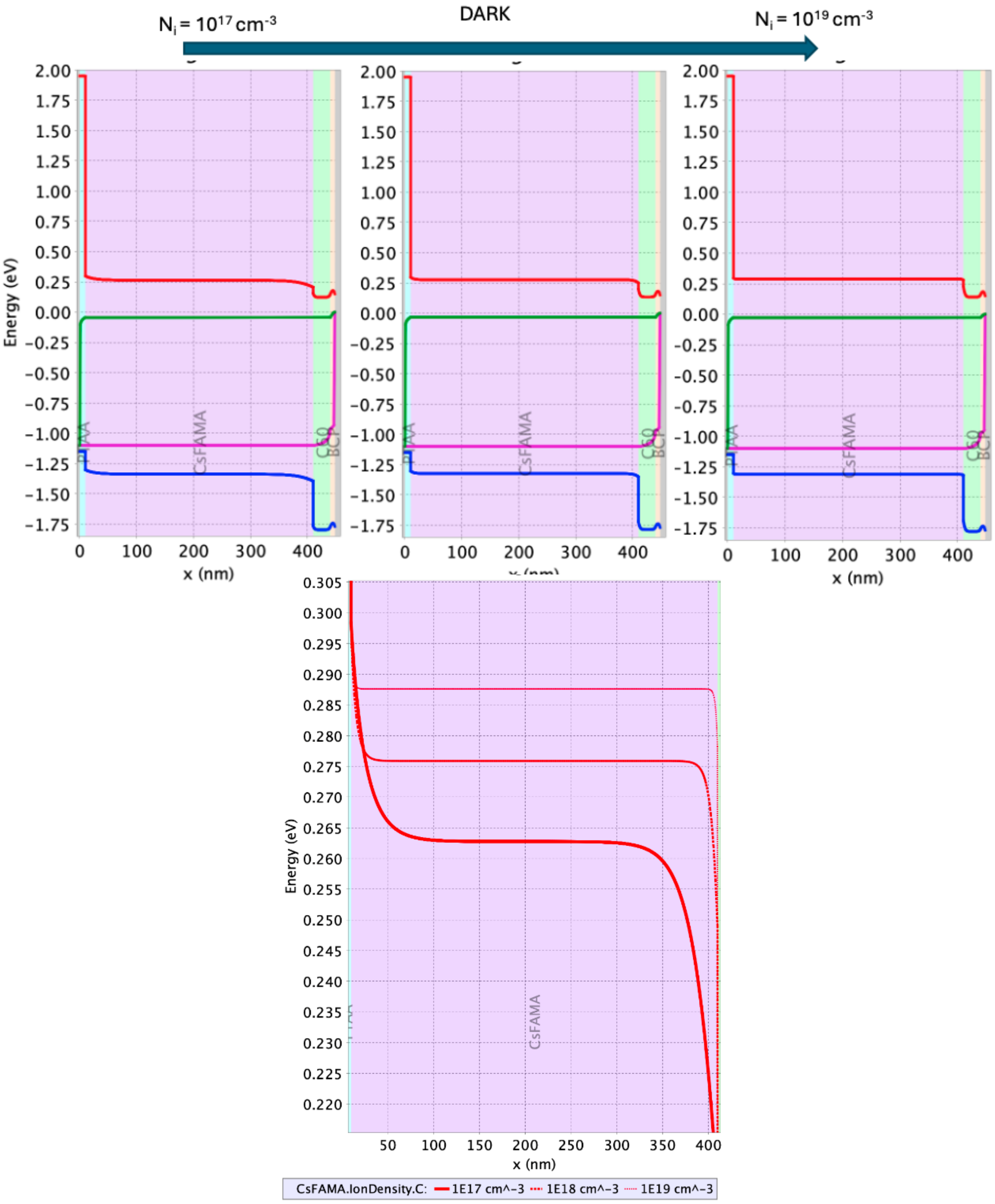

Figure S6. Simulated energy levels with varying densities of ions (N_i). It can be observed how the band bending decreases as the ionic density increases. Red and blue lines stand for the LUMO and HOMO levels, respectively. Green and magenta lines are the quasi-Fermi levels for electrons and holes respectively. Note that a stronger screening of the electric field on the *perovskite side* occurs because of the larger concentration of ions (cations) and subsequent accumulation at the interface (Figure S4).

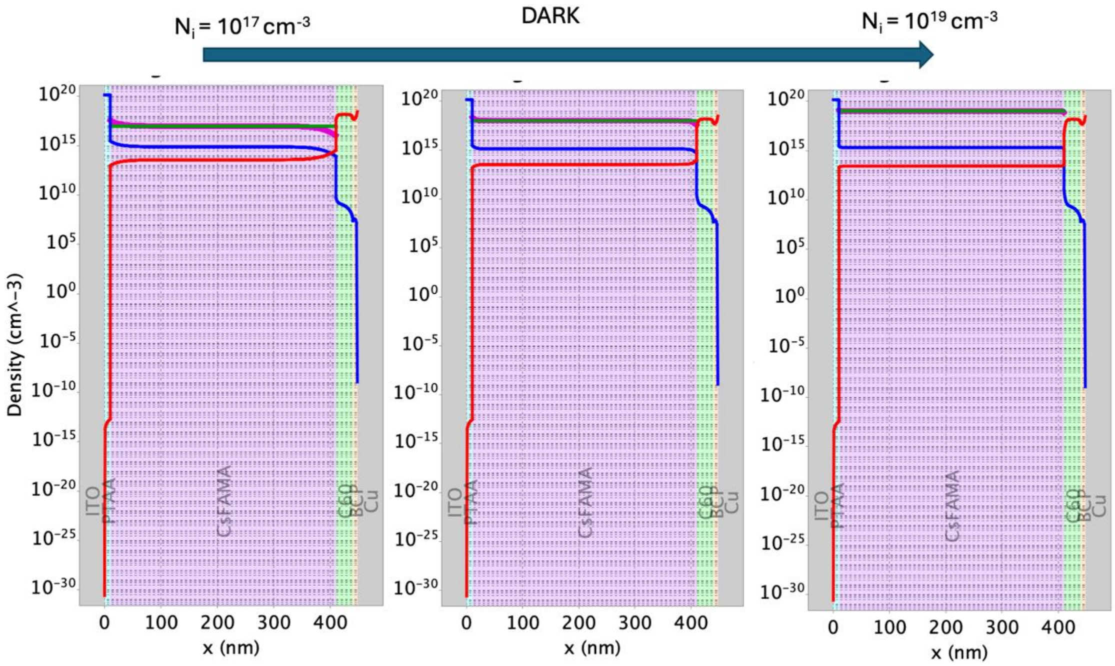

Figure S7. Simulated evolution of the charge density profiles with the variation of ionic density. Code color: red (electrons), blue (holes), green (immobile anions), magenta (mobile cations). Observe how the width of the double layer (cation and hole accumulation) decreases as the ionic density increases.

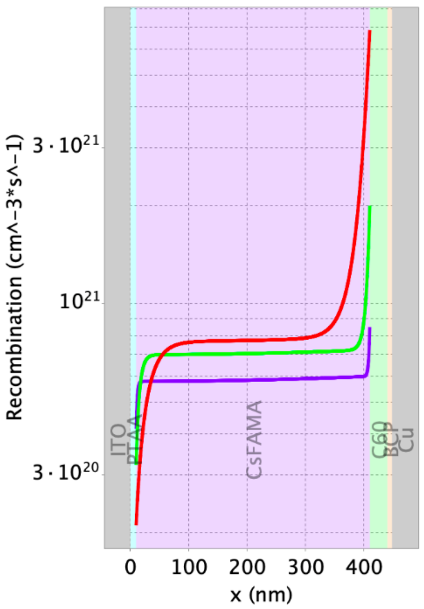

Figure S8. Recombination rate profiles for varying values of the ion density (10^{17} cm^{-3} red, 10^{18} cm^{-3}, green, 10^{19} cm^{-3}, violet). Note that the recombination rate significantly decreases near the PTAA surface, showing a reduction by approximately a factor of six for the lowest ion density studied. This reduction becomes less pronounced as the ion concentration increases, with the recombination rate gradually approaching the bulk value at shorter distances from the surface. This behaviour aligns with the mechanism described in Figure 5, which focuses on the interfacial dynamics.

R_L-L line effect on the EC model used in this work.[1]

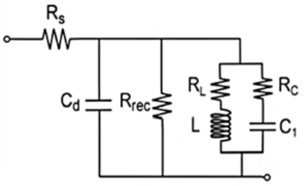

In this model, surface ion accumulation causes a delay, which is defined by the kinetic relaxation time. This delay, like the inductance, resists current changes. This delayed process leads to low-frequency arcs and a negative capacitance, effectively represented by the inductor.[2] The ionic movement also creates an additional recombination pathway (R_L). Without ion accumulation, the primary recombination pathway is through R_{rec}.
The AC impedance response corresponding to this model can be expressed as follows:

$$Z = \frac{\hat{V}}{\hat{j}} = \left[i\omega C_d + \frac{1}{R_L + i\omega L} + \frac{1}{R_{rec}} + \frac{1}{R_C - i\frac{1}{\omega C_1}} \right]^{-1} \quad \text{(S1)}$$

$$R_{rec} = \frac{\beta k_B T}{q\bar{J}_{rec}} \quad \text{(S2)}$$

$$R_L = \frac{\gamma k_B T}{q\bar{j}_{rec}} \quad \text{(S3)}$$

$$L = \frac{R_L}{\tau_{kin}} \quad \text{(S4)}$$

$$R_C = \frac{b\tau_{kin}}{C_1} \quad \text{(S5)}$$

Where j_{rec} is the recombination current at steady state, β and γ exponents are constants with values ≤ 1 and *b* is a correction factor for the fitting, with k_BT as the thermal energy. We would like to remark that parameter b is just a correction factor that does not play any crucial role in the obtention of the results and conclusions of this work.
The inclusion of the R_C-C_1 branch is based on the surface polarization model (Ref 1), which effectively captures the behavior of the PTAA sample. In contrast, the SAM device is more accurately described just by the R_L-L branch at low frequencies, aligning with the chemical inductor model recently proposed by Bisquert and Guerrero.[3]
The surface polarization model explains how charge accumulation at the interface between the active layer and the charge transport layer creates a polarization effect that significantly influences capacitance and resistance values. The branch Rc-C_1 introduces the capacitive behavior from interfacial polarization, and the R_L-L branch introduces the inductive effect, indicative of ionic dynamics.
On the other hand, the chemical inductor model explores the origin of the inductive behavior. This model attributes this phenomenon to ionic migration and its interaction with charge recombination processes at the interface. It establishes a link between the inductive response and the presence of mobile ions, highlighting how these dynamics influence recombination rates.
As mentioned in the main text, recent advancements in the Surface Polarization Model (SPM) have led to the development of the Modified Surface Polarization Model (m-SPM)[4] which introduces more refined assumptions for a more accurate description of charge transport. Specifically, the m-SPM accounts for the influence of electronic carriers, particularly holes, within the perovskite layer and incorporates a nonlinear potential

evolution, providing a more realistic framework for understanding hysteresis effects in perovskite solar cells (PSCs). While the m-SPM offers a more precise theoretical foundation, the use of the original SPM in this study does not affect the validity of our conclusions. The results remain well-supported by drift-diffusion (DD) simulations, demonstrating consistency between different analytical approaches. In particular, the trend of τ_{kin}, obtained from the fitting using the equivalent circuit (EC) from the standard SPM, aligns well with the time constant directly extracted from the impedance spectra (Cole-Cole plot). This agreement confirms that the original SPM provides a reliable framework for interpreting the observed charge dynamics, particularly in distinguishing the slower ionic processes in PTAA-based devices compared to the more stable behavior observed in SAM-based devices.

Impedance response during the electroformation process in a perovskite-based memristor:

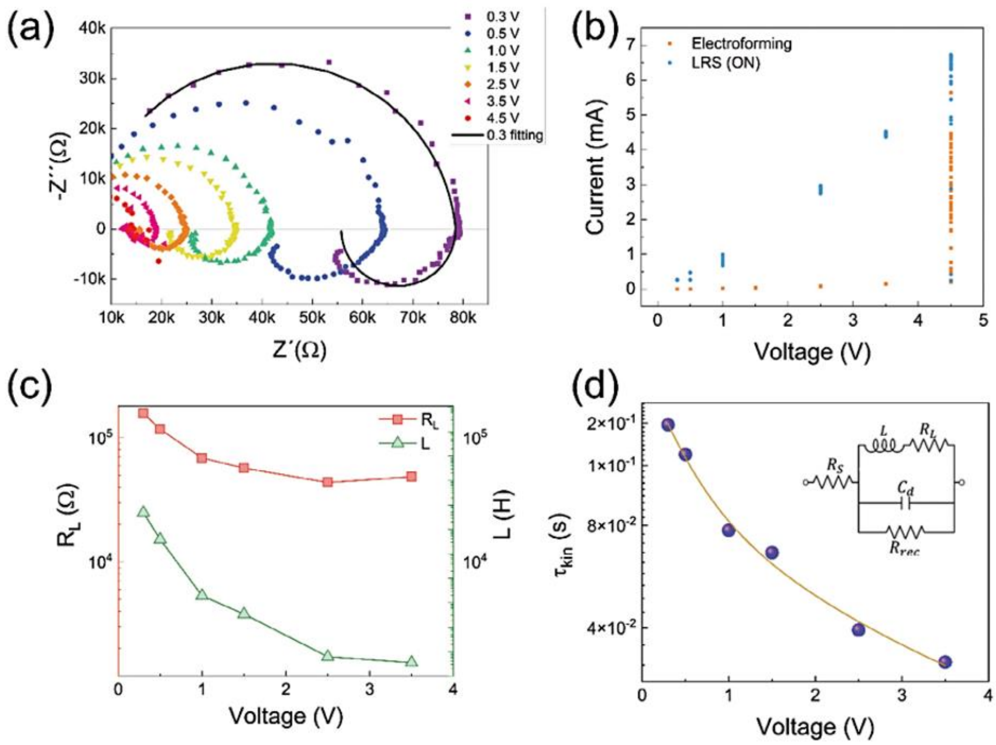

Figure S8. (a) Evolution of the impedance response (dark conditions) with the applied bias leading to the electroforming process. Note the reduction of the HF arc with the increasing voltage approaching the LRS. (b) Related DC extracted from the IS during the electroforming process (orange dots) and LRS (blue dots). (c) R_L and L fitting parameters from the EC (inset Fig. S5d). (d) Kinetic relaxation time (τ_{kin}) evolution during the electroforming process. Note the change in the ionic dynamic from the accumulation regime to ion diffusion (a faster process). Reproduced with permission of Ref. 31.

References

1. Ravishankar, S.; Almora, O.; Echeverría-Arrondo, C.; Ghahremanirad, E.; Aranda, C.; Guerrero, A.; Fabregat-Santiago, F.; Zaban, A.; Garcia-Belmonte, G.; Bisquert, J. Surface Polarization Model for the Dynamic Hysteresis of Perovskite Solar Cells. *J Phys Chem Lett* **2017**, *8* (5), 915–921. https://doi.org/10.1021/acs.jpclett.7b00045.
2. Ghahremanirad, E.; Bou, A.; Olyaee, S.; Bisquert, J. Inductive Loop in the Impedance Response of Perovskite Solar Cells Explained by Surface Polarization Model. *J Phys Chem Lett* **2017**, *8* (7), 1402–1406. https://doi.org/10.1021/acs.jpclett.7b00415.
3. Bisquert, J.; Guerrero, A. Chemical Inductor. *J Am Chem Soc* **2022**, *144* (13), 5996–6009. https://doi.org/10.1021/jacs.2c00777.
4. Clarke, W.; Cowley, M. V; Wolf, M. J.; Cameron, P.; Walker, A.; Richardson, G. Inverted Hysteresis as a Diagnostic Tool for Perovskite Solar Cells: Insights from the Drift-Diffusion Model. *J Appl Phys* **2023**, *133* (9), 095001. https://doi.org/10.1063/5.0136683.
