# Supplementary material for: Insights from Impedance Spectroscopy in Perovskite Solar Cells with Self-Assembled Monolayers: Decoding SAM’s Tricks
Source: J Phys Chem Lett. 2025 Feb 24;16(9):2301–8. doi: 10.1021/acs.jpclett.4c03194 (PMC12142668; doi:10.1021/acs.jpclett.4c03194)
Supplement: Supplementary file 2 [file jz4c03194_si_002.pdf]

jz-2024-031946.R1

Name: Peer Review Information for "Insights from Impedance Spectroscopy in Perovskite Solar Cells with Self-Assembled Monolayers: Decoding SAM´s Tricks"

First Round of Reviewer Comments

Reviewer: 1

Comments to the Author

The use of self-assembled monolayers (SAMs) is considered as an effective approach for interface engineering in perovskite solar cells. In this paper, the authors study the effect of adding a SAM layer on the impedance response of perovskite solar cells compared to the classical behavior shown by photovoltaic devices with, for example, PTAA.

First of all, I think the title is too exaggerated. The authors study a perovskite structure with one type of SAM, are the conclusions reached here universal? Next, it is confusing to the reader that Figure 1 shows current-voltage curves with no hysteresis at 100 mV/s and Figure 2 suggests prominent hysteresis at that sweep voltage speed. In Figure 2c, were no steady-state curves (HI=0) obtained? Here, moreover, a diffuse trend of hysteresis is observed, suggesting either that the measurements are not controlled or that the HI may not quantify too accurately the evolution of the current-voltage curves. As for impedance, I think the analysis is not deep enough. First of all, I don't understand why in the text the spectra are shown in dark and none are shown under illuminated conditions. In all the impedance spectra, three processes are, more or less, sharply visualized. How do the authors interpret this when, in general, there is a strict equality between the slow time constants (RC and RL branches of the circuit shown in SI)? In this sense, there are many recent studies studying the impedance of perovskite solar cells that the authors have ignored. I understand that the authors “play” with the value of the parameter b of the model, but there is no reference to it, nor any physical significance in relation to the nonlinear model that is the genesis of the impedance. This certainly needs to be re-explained.

Furthermore, several of the numerical fittings shown throughout the paper do not suggest a high accuracy in the agreement between experimental and simulated data (Figs. 3 and S6), which could invalidate some conclusions. How do the authors explain capacitance values of 1e-11 uF (see figure S1)? On the other hand, I would like to indicate that Figure 5 of the paper is very similar to one already shown in a recent publication, j.joule.2023.11.011, which makes me concerned about replication of similar studies. Finally, following the SI is very difficult as there are figures that are

not… For all these reasons, I think that the paper should be rejected or reconsidered in a sister journal.

Reviewer: 2

Comments to the Author

The manuscript by Aranda et al, presents an impedance spectroscopy study of pin solar cells using SAMs as hole transport layers. The work discusses the role of SAMs in enhanced Voc and suppressed surface recombination in perovskite solar cells. The article presents important insights regarding the mechanisms that make SAMs a popular choice for HTLs in several reported perovskite solar cells. Overall, the conclusion is that SAMs reduce the ion concentration at the interface. The work is interesting and can be considered for publication after the following minor points:

1. Page 4, first paragraph. The sentence: 'The enhancement in the device performance cannot be linked to the HOMO level of the SAM, as PTAA has a deeper HOMO level'. Could the authors add the respective references where the HOMO levels of the EADR03 SAMs are reported?

2. Can the results be extended to other SAMs? E.g. 2PACz, MeO-4PACz, etc?

3. Page 4, line 48-49: ' … ITO is mainly based on In2O3 doped with 20% Sn …' , please correct if it is 20 at% Sn or 20 wt% SnO2?

4. Devices from Fig. 2. Do the devices undergo identical processing conditions? Is the perovskite growth influenced by the substrate? And are the SAMs assumed to be one monolayer (in some cases called washed) or multiple layers? if not a monolayer (i.e. uncontrolled thickness), how would this affect ion accumulation?

5. Figure 2c. Is the point at the scan rate of 10^2 mVs-1 for PTAA a reproducible effect? Can the authors present some statistical distribution in the points presented?

6. XPS results. How does the thickness of the HTL influences the quantification of the OH- signals?

7. Are the SAMs or PTAA annealed? An annealing step for the HTL would also modify the concentration of OH-. Please comment.

Reviewer: 3

Comments to the Author

The manuscript provides a comprehensive investigation of self-assembled monolayers (SAMs) as hole-transport layers (HTLs) in p-i-n perovskite solar cells, emphasizing their role in improving device performance and stability. The work employs a range of experimental techniques, including impedance spectroscopy (IS), X-ray photoelectron spectroscopy (XPS), and numerical simulations, to elucidate the mechanisms underlying the benefits of SAMs. The claimed findings would be significant and add valuable insights to the field of perovskite photovoltaics, particularly in addressing interface-related issues such as ion migration and surface recombination.

However, I think that the conclusions extracted from the experimental findings are too ambitious, the discussion of the impedance spectroscopy results is not fully correct, and the simulations do not support the claims of the authors.

I will point out two primary concerns argue against the publication of this article:

1. The main result and the conclusions from it are claimed to be extracted from impedance spectroscopy, as indicated in the title of the paper. However, the difference in the characteristic time tau_k from the fresh samples is not even an order of magnitude great. In the case of the aged samples, we can see in the SI that the inductive loop of the PTAA device is not a clear arc and it's more of a concatenation of noisy points, therefore, the characteristic time extracted from it is unreliable. The whole paper is supported in this difference, therefore, this experiments need more clarification. Moreover, the PTAA sample has an additional arc in respect to the SAM sample, and this could hinder the extraction of the time constant. The authors don't comment on that.

2. The drift-diffusion simulation do not support the mechanism proposed in Figure 5. In fact, they do not show more band bending (they show less) with increasing number of ions; they don't show more ions in the interface either; and they show more recombination with fewer ions. Additionally, the simulations increase the number of ions generally, and not the accumulation in one interface, which is not the mechanism that the authors claim. Therefore, this simulations would not support the hypothesis either. This referee is aware of the difficulty of simulating such a phenomenon, but the present simulations do not support the mechanism the authors claim.

Among other errors, the authors say that the hysteresis decreases with increasing scan rates, because the HI is decreasing, but it is actually an increase of inverted hysteresis.

In light of these concerns, I believe the manuscript requires significant revision to adequately support its claims.

Author's Response to Peer Review Comments:

The response to referees is attached as a docx file.

02-Dec-2024

Journal: The Journal of Physical Chemistry Letters
Manuscript ID: jz-2024-031946
Original Submission Date: 05-Nov-2024
Title: "Unveiling SAM's Tricks: Impedance Spectroscopy Analysis of Self-Assembled Monolayers-Based Perovskite Solar Cells"
Author(s): Aranda, Clara; Li, Wenhui; Martinez-Ferrero, Eugenia; Pistor, Paul; Oskam, Gerko; Palomares, Emilio; Anta, Juan

Reviewer(s)' Comments to Author:

Reviewer: 1

Recommendation: Reconsider as an article in The Journal of Physical Chemistry A/B/C.

Comments:
The use of self-assembled monolayers (SAMs) is considered as an effective approach for interface engineering in perovskite solar cells. In this paper, the authors study the effect of adding a SAM layer on the impedance response of perovskite solar cells compared to the classical behavior shown by photovoltaic devices with, for example, PTAA.

First of all, I think the title is too exaggerated. The authors study a perovskite structure with one type of SAM, are the conclusions reached here universal?

We sincerely thank the referee for their valuable input. While our study focuses on a specific type of SAM, the mechanisms identified here and previous findings by the authors on surface treatments and ionic immobilization, strongly suggest that these processes are universal in driving the enhanced performance of perovskite solar cells incorporating SAMs.

Our results can likely be generalized to other SAMs, as they share a common mechanism of forming ordered arrays on the ITO substrate via covalent bonding between their carboxylic or phosphonic acid anchoring groups and the hydroxyl groups on the ITO surface. These covalent bonds effectively mitigate the accumulation of positive charges at the interface, thereby reducing surface recombination, a benefit that we anticipate would extend to other SAMs as well.

Nevertheless, we have modified the title for better accuracy: *Insights from Impedance Spectroscopy in Perovskite Solar Cells with Self-Assembled Monolayers: Decoding SAM´s Tricks*

Next, it is confusing to the reader that Figure 1 shows current-voltage curves with no hysteresis at 100 mV/s and Figure 2 suggests prominent hysteresis at that sweep voltage speed. In Figure 2c, were no steady-state curves (HI=0) obtained? Here, moreover, a diffuse trend of hysteresis is observed, suggesting either that the measurements are not controlled or that the HI may not quantify too accurately the evolution of the current-voltage curves.

We appreciate the referee's comment and understand the potential for perceived incongruence. However, we would like to clarify that the samples shown in Figure 1 represent the champion cells previously reported in our earlier work, as cited in the text. In contrast, the cells used for the impedance analysis in Figure 2 were not champion cells. These measurements were conducted in a different laboratory after the cells were transported and subjected to conditions different from those experienced by the champion cells.

Specifically, variations in temperature, humidity, and light source could have influenced the behaviour of the cells, contributing to the observed differences.

The primary aim of the hysteresis analysis is to emphasize the distinctions between the performance of SAM-containing cells and those using the conventional PTAA layer. As noted in our prior publication (https://doi.org/10.1021/acs.jpclett.0c02331), significant variations in the hysteresis index, such as the trend toward inverted hysteresis with changes in scan rate, serve as indicators of interfacial phenomena that may lead to undesirable recombination processes. The hysteresis data presented here reinforce the observation that the PTAA/perovskite interface behaves distinctly from the SAM-containing interface.

Additionally, we would like to underscore that the hysteresis index for SAM-based cells at a scan rate of 100 mV/s, as shown in Figure 2, remains below 5%, while the HI of PTAA has gone to a strong inverted hysteresis, which again, highlight the differences occurring at the interfaces of SAM/perovskite and PTAA/perovskite respectively.

We have however modified the text in the manuscript for better accuracy in our data presentation:

It is important to note that for the analysis of the electronic responses (CV and IS), the cells were transported to a different laboratory, exposing them to different conditions than those experienced by the champion cells shown in Figure 1.

(...)Inverted hysteresis, where the efficiency obtained from the forward scan exceeds that of the reverse scan, is more pronounced in the case of the PTAA device. As discussed in our previous publication[20], significant trends toward inverted hysteresis serve as indicators of interfacial phenomena that may lead to undesirable recombination processes. The hysteresis data presented here reinforce the observation that the PTAA/perovskite interface is more affected by recombination mechanisms compared to the SAM-containing interface.

The evolution of the HI with the scan rate observed for both samples also aligns with the work reported by García-Rodríguez and coworkers.[21] They combined experimental and modelling approaches to demonstrate the dependence of hysteresis on scan rate. The cell configuration (p-i-n, or n-i-p), the ion diffusion coefficient, and the nature of charge transport layers determine the crossover from normal to inverted hysteresis. These observations reflect the distinct nature of the HTL materials in our study and highlight their influence on hysteresis behaviour.

As for impedance, I think the analysis is not deep enough. First of all, I don't understand why in the text the spectra are shown in dark and none are shown under illuminated conditions.

We thank the referee for their input and appreciate the opportunity to clarify this point. Impedance spectroscopy can indeed be conducted under various conditions, depending on the specific information being sought after. If the goal is to assess charge extraction capability, measurements should be performed at short circuit (V = 0). However, in our case, the focus is on understanding the recombination processes within the system, as these are directly related to the observed improvements in V_{oc} for the SAM samples. Since V_{oc} is inherently linked to recombination, it is most appropriate to conduct the impedance measurements near open circuit conditions.

As the referee may know, impedance measurements must be performed under steady-state conditions to ensure reliable data. This is achieved by tracking the DC current during the application of different frequencies and, ideally, conducting chronoamperometry before, during, and after the measurements. When measurements are conducted under illumination, particularly over the wide frequency range needed to account for ionic contributions (including very low frequencies), the extended measurement duration can

accelerate degradation processes in the samples. While this may not significantly affect the more stable SAM-based devices, it is likely to impact the PTAA samples, potentially leading to unreliable results.

To ensure consistent and reliable conditions for both types of devices, we chose to perform the impedance measurements in the dark, injecting carriers at 1.1 V, which is close to the V_{oc} conditions. Measurements under these conditions provide similar insights into the recombination processes as those conducted under illumination at V_{oc}, while minimizing the risk of degradation during the measurement. We also wish to emphasize that performing impedance spectroscopy under dark conditions is a common approach in the literature for perovskite solar cell analysis, precisely for the reasons outlined above. (https://doi.org/10.1021/acs.jpclett.0c02331, https://doi.org/10.1063/1.4966127, https://doi.org/10.1021/acs.jpclett.5b00480)

Nevertheless, we have included this explanation in the main text to avoid unclarity:

This work focuses on understanding the recombination processes within the system, as they are directly linked to the observed improvements in open-circuit voltage (V_{oc}) for the SAM samples. Given that V_{oc} is inherently tied to recombination, conducting impedance measurements near open-circuit conditions is essential.
However, when measurements are performed under illumination, especially across a wide frequency range needed to capture ionic contributions (including very low frequencies down to 10 mHz), the extended duration of such measurements can accelerate degradation in the samples. To ensure consistent and reliable conditions for both types of devices, we opted to conduct the impedance measurements in the dark while injecting carriers at 1.1 V, which closely approximates the V_{oc} conditions. These measurements provide similar insights into the recombination processes as those taken under illumination at V_{oc}, but with the added benefit of minimizing degradation risks during the process.

In all the impedance spectra, three processes are, more or less, sharply visualized. How do the authors interpret this when, in general, there is a strict equality between the slow time constants (RC and RL branches of the circuit shown in SI)? In this sense, there are many recent studies studying the impedance of perovskite solar cells that the authors have ignored.

We appreciate the referee's comment, but we are uncertain about the meaning of the phrase "strict equality between the slow time constants." As the referee may observe, the impedance responses of the PTAA and SAM devices are distinctly different. Specifically, the PTAA device exhibits an additional arc in the impedance spectrum, corresponding to the Rc-C1 branch in the equivalent circuit (EC). This arc is absent in the SAM device, which is the key distinction requiring us to include the Rc-C1 branch to fit the PTAA data, but not the SAM data.

The inclusion of the Rc-C1 branch is based on the surface polarization model(https://doi.org/10.1021/acs.jpclett.7b00045), which accurately reflects the behaviour of the PTAA sample. In contrast, the SAM device is better described using only the RL-L branch at low frequencies, consistent with the chemical inductor model recently reported by Bisquert and Guerrero (https://doi.org/10.1021/jacs.2c00777). To improve clarity, we have provided a more detailed explanation of these models in the Supporting Information:

The inclusion of the R_C-C1 branch is based on the surface polarization model (Ref 1), which effectively captures the behavior of the PTAA sample. In contrast, the SAM device is more accurately described just by the R_L-L branch at low frequencies, aligning with the chemical inductor model recently proposed by Bisquert and Guerrero (https://doi.org/10.1021/jacs.2c00777).

The surface polarization model explains how charge accumulation at the interface between the active layer and the charge transport layer creates a polarization effect that significantly influences capacitance and resistance values. The branch Rc-C1 introduces the capacitive behavior from interfacial polarization, and the RL-L branch introduces the inductive effect, indicative of ionic dynamics.

On the other hand, the chemical inductor model explores the origin of the inductive behavior. This model attributes this phenomenon to ionic migration and its interaction with charge recombination processes at the interface. It establishes a link between the inductive response and the presence of mobile ions, highlighting how these dynamics influence recombination rates.

The co-existence of these two branches in the EC representing the behavior of the PTAA impedance response implies that PTAA devices suffer from greater recombination losses, with both electronic interfacial phenomena and ionic effects contributing to performance degradation.

Given these distinctions and the use of separate physical models to interpret the branches, we do not understand the referee's point regarding "strict equality," as this does not apply to our analysis.

Finally, we agree with the referee that there is a vast amount of recent literature on the interpretation of impedance spectra for perovskite solar cells. However, we believe to have cited all relevant studies necessary for this work. In any case, we have added a couple of additional references to address the referee's request.

I understand that the authors "play" with the value of the parameter b of the model, but there is no reference to it, nor any physical significance in relation to the nonlinear model that is the genesis of the impedance. This certainly needs to be re-explained.

We would like to clarify that parameter *b* does not carry any physical meaning; it serves solely as a mathematical correction factor for the fitting process (as mentioned in the text on page 6 of the supporting information). While it is true that an impedance spectrum can be fitted using various equivalent circuits (ECs), this does not necessarily ensure accuracy in terms of the underlying physics.

In our case, the choice and use of fitting correction factors do not alter the physical interpretation or validity of our analysis. The fitting approach was carefully selected to provide meaningful insights into the physical processes occurring within the system, ensuring that the analysis remains robust and reliable.

Nevertheless, we have included two additional figures presenting the bode plots of the impedance response, (see below) of both PTAA and SAM aged and fresh devices to confirm the differences in the time constants.

Furthermore, several of the numerical fittings shown throughout the paper do not suggest a high accuracy in the agreement between experimental and simulated data (Figs. 3 and S6), which could invalidate some conclusions.

We agree with the referee's observation that the experimental data is not perfectly replicated by the model. However, achieving a perfect fit was not the objective of our modelling studies, as this would require a systematic and detailed mapping of all parameters involved. Instead, a simplified drift-diffusion model was designed to capture and replicate the essential overall trends observed in the experimental data. Notably, the model successfully reproduces the two arcs observed in the first and second quadrants, which correlate with two peaks of opposite signs in the frequency plot.

Furthermore, the frequency positions of these signals align very well with the experimental data obtained in the dark. While the width of the arcs (low and high-frequency resistances) shows some deviation from the experimental results, this can likely be attributed to a mismatch between the internal voltage and the

applied voltage. It is well-documented that the size of the arcs is highly sensitive to the applied voltage (see, for example, 10.1039/C9TA02808K; 10.1016/j.nanoen.2018.03.042).

We would also like to highlight that the drift-diffusion model employed here has been successfully applied in previous studies to interpret impedance data of perovskite solar cells under both illuminated and dark conditions (10.1103/PhysRevApplied.19.014061; 10.1039/D0NR03058A; 10.1039/D2TA04840J; 10.1063/5.0216983).

How do the authors explain capacitance values of 1e-11 uF (see Figure S1)? On the other hand, I would like to indicate that Figure 5 of the paper is very similar to one already shown in a recent publication, j.joule.2023.11.011, which makes me concerned about the replication of similar studies. Finally, following the SI is very difficult as there are figures that are not... For all these reasons, I think that the paper should be rejected or reconsidered in a sister journal.

We thank the referee for his/her observation. That value is in fact a typo, the scale should be in F and not in μF. We have modified the label in the graph.

Regarding the comment on Figure 5, we acknowledge that similarities between figures in different studies by the same authors can occur. However, this does not imply that the studies are replicas of each other. On the contrary, the similarities reflect consistency and coherence in the research line, with each study providing robust and reliable data that support and build upon one another.

Specifically, the analysis of V_{oc} and the exploration of interfacial effects and mechanisms underlying improvements in this key photovoltaic parameter are central to the research goals of the authors. For this reason, the findings presented in this manuscript are supported by prior works, including the study published in *Joule*, as well as related investigations on negative capacitance/ inverted hysteresis, and perovskite memristors (both already cited in the text).

Rather than being a replication, this manuscript represents a follow-up study that advances the same research trajectory, strengthening the reliability and robustness of the findings across multiple works.

Considering the comment about the SI, we have implemented changes that we believe have improved the legibility and clarity of the presented data.

Additional Questions:
Urgency: Moderate
Significance: Moderate
Novelty: Moderate
Scholarly Presentation: Low

Is the paper likely to interest a substantial number of physical chemists, not just specialists working in the authors' area of research?: No

Reviewer: 2

Recommendation: This paper is publishable subject to minor revisions noted. Further review is not needed.

Comments:
The manuscript by Aranda et al, presents an impedance spectroscopy study of pin solar cells using SAMs as hole transport layers. The work discusses the role of SAMs in enhanced Voc and suppressed surface

recombination in perovskite solar cells. The article presents important insights regarding the mechanisms that make SAMs a popular choice for HTLs in several reported perovskite solar cells. Overall, the conclusion is that SAMs reduce the ion concentration at the interface. The work is interesting and can be considered for publication after the following minor points:

We appreciate the positive feedback from the reviewer and the suggestions to improve the quality of the manuscript.

1. Page 4, first paragraph. The sentence: 'The enhancement in the device performance cannot be linked to the HOMO level of the SAM, as PTAA has a deeper HOMO level'. Could the authors add the respective references where the HOMO levels of the EADR03 SAMs are reported?

We thank the referee for the suggestion. We have added a reference, already included in the manuscript in which we estimated the HOMO levels by UPS measurements (Reference 15: *Energy Environ. Sci., 2021, 14, 3976-3985*). The results indicated that the HOMO level of PTAA (-5.2 eV) was deeper than that of SAM (-5.05 eV).

2. Can the results be extended to other SAMs? E.g. 2PACz, MeO-4PACz, etc?

See also answer 1 to referee 1. We believe that the results from this study can be extrapolated to other SAMs. The SAMs share a common mechanism to form ordered arrays onto the ITO substrate through the formation of covalent bonding between the carboxylic or phosphonic acids of the anchoring groups with the -OH groups present on the ITO surface. The formation of these bonds diminishes the accumulation of positive charges at the interface; therefore, the effect of the formation of SAMs on the reduction of surface recombination is expected to be observed when other SAMs are used.
We appreciate the question of the referee, and we have included a comment on the Conclusions, which reads as follows:

"This effect is expected to be observed in other SAMs that chemically bond to the ITO surface. Therefore, this manuscript just provides more fundamental knowledge to understand the improvement seen on SAM-based devices."

3. Page 4, line 48-49: ' … ITO is mainly based on In2O3 doped with 20% Sn …', please correct if it is 20 at% Sn or 20 wt% SnO2?

We have corrected it in the manuscript to indicate that this corresponds to 20 wt% SnO_2.

4. Devices from Fig. 2. Do the devices undergo identical processing conditions? Is the perovskite growth influenced by the substrate? And are the SAMs assumed to be one monolayer (in some cases called washed) or multiple layers? if not a monolayer (i.e. uncontrolled thickness), how would this affect ion accumulation?

Thanks for the comments. We will answer the questions individually:

i) Devices from Fig. 2. Do the devices undergo identical processing conditions?

Yes, to ensure a reliable comparison study, the devices undergo identical processing conditions and identical measuring processes.

ii) Is the perovskite growth influenced by the substrate?

Yes, the wettability of the modified substrate can affect the crystal growth of the perovskite at the nanometric scale, resulting in differences in crystal quality, grain size and morphology

iii) And are the SAMs assumed to be one monolayer (in some cases called washed) or multiple layers? if not a monolayer (i.e. uncontrolled thickness), how would this affect ion accumulation?

The hole-selective contact composed of self-assembled molecules is an ultra-thin layer with an estimated thickness of around 2-3 nm which is close to the length of the molecules. The control of the thickness of the SAM layer is achieved using diluted solutions of SAMs for the deposition by spin coating (we normally use 0.1 mM concentration).
In any case, the ion accumulation at the interface is promoted by the presence of -OH groups from the ITO. The SAMs chemically react with the hydroxyl groups consequently reducing their concentration. Therefore, in the event of an uncontrolled thickness, that could be of just a few nanometers, we expect that the ion accumulation will be reduced anyway.

5. Figure 2c. Is the point at the scan rate of 10^2 mVs-1 for PTAA a reproducible effect? Can the authors present some statistical distribution in the points presented?

We thank the referee for highlighting this point. Unfortunately, we do not have sufficient statistical data to confirm it as a reproducible effect. However, this observation is indeed very interesting and will be considered in future studies.

6. XPS results. How does the thickness of the HTL influences the quantification of the OH- signals?

The sensitivity of the XPS technique allows the study of the top 10 nm of the material (see, for example, the description of the technique provided at https://www.thermofisher.com/us/en/home/materials-science/xps-technology.html).

In this work, the thickness of the PTAA film is estimated to be 10 nm, while that of SAM is estimated to be 2-3 nm, which means that the detection of the OH- signal by XPS is reliable.

7. Are the SAMs or PTAA annealed? An annealing step for the HTL would also modify the concentration of OH-. Please comment.

We thank the referee for this interesting observation. We agree that the annealing step can help to remove the -OH on the ITO surface. However, in our work, the PTAA is annealed at 100 °C for 10 min after spin-coating, while the deposition of the SAM is achieved without post-annealing. The O 1s signal obtained from XPS analysis suggests that the -OH groups on the ITO/SAM substrate are significantly reduced in comparison to the ITO/PTAA substrate, further supporting that the reduction of -OH in the ITO/SAM substrate is due to the reaction between the SAM and the -OH groups and not to any annealing processing.

Additional Questions:

Urgency: Moderate
Significance: High
Novelty: Moderate
Scholarly Presentation: High

Is the paper likely to interest a substantial number of physical chemists, not just specialists working in the authors' area of research?: Yes

Reviewer: 3

Recommendation: Reconsider as an article in The Journal of Physical Chemistry A/B/C.

Comments:
The manuscript provides a comprehensive investigation of self-assembled monolayers (SAMs) as hole-transport layers (HTLs) in p-i-n perovskite solar cells, emphasizing their role in improving device performance and stability. The work employs a range of experimental techniques, including impedance spectroscopy (IS), X-ray photoelectron spectroscopy (XPS), and numerical simulations, to elucidate the mechanisms underlying the benefits of SAMs. The claimed findings would be significant and add valuable insights to the field of perovskite photovoltaics, particularly in addressing interface-related issues such as ion migration and surface recombination.

However, I think that the conclusions extracted from the experimental findings are too ambitious, the discussion of the impedance spectroscopy results is not fully correct, and the simulations do not support the claims of the authors.

I will point out two primary concerns argue against the publication of this article:

1. The main result and the conclusions from it are claimed to be extracted from impedance spectroscopy, as indicated in the title of the paper. However, the difference in the characteristic time tau_k from the fresh samples is not even an order of magnitude great. In the case of the aged samples, we can see in the SI that the inductive loop of the PTAA device is not a clear arc and it's more of a concatenation of noisy points, therefore, the characteristic time extracted from it is unreliable. The whole paper is supported in this difference, therefore, this experiments need more clarification. Moreover, the PTAA sample has an additional arc in respect to the SAM sample, and this could hinder the extraction of the time constant. The authors don't comment on that.

We thank the referee for their observations and the opportunity to clarify. We would like to emphasize that the impedance responses represented in Figure S2 are not the sole supporting evidence of our work. While we acknowledge the referee’s point about the noisy low-frequency response in the PTAA sample, this observation underscores the lower stability of PTAA samples compared to SAM samples.

Additionally, the hysteresis analysis at different scan rates demonstrates an increase in inverted hysteresis in the PTAA sample. This aligns with our previous findings, where pronounced inverted hysteresis correlates with negative capacitance and is associated with undesirable recombination mechanisms that negatively impact V_{oc}.

Moreover, the XPS analysis supports our conclusions by showing that the presence of SAM reduces the amount of hydroxyl groups (-OH) at the surface, thereby preventing cation accumulation at the interface and mitigating recombination processes.

To further strengthen our argument, we invite the referee to review the Bode plot figures now added to the Supporting Information (Figure S3). These plots clearly illustrate the evolution of time constants from fresh to aged devices for both PTAA and SAM samples, highlighting distinct differences even before considering the fitting results.

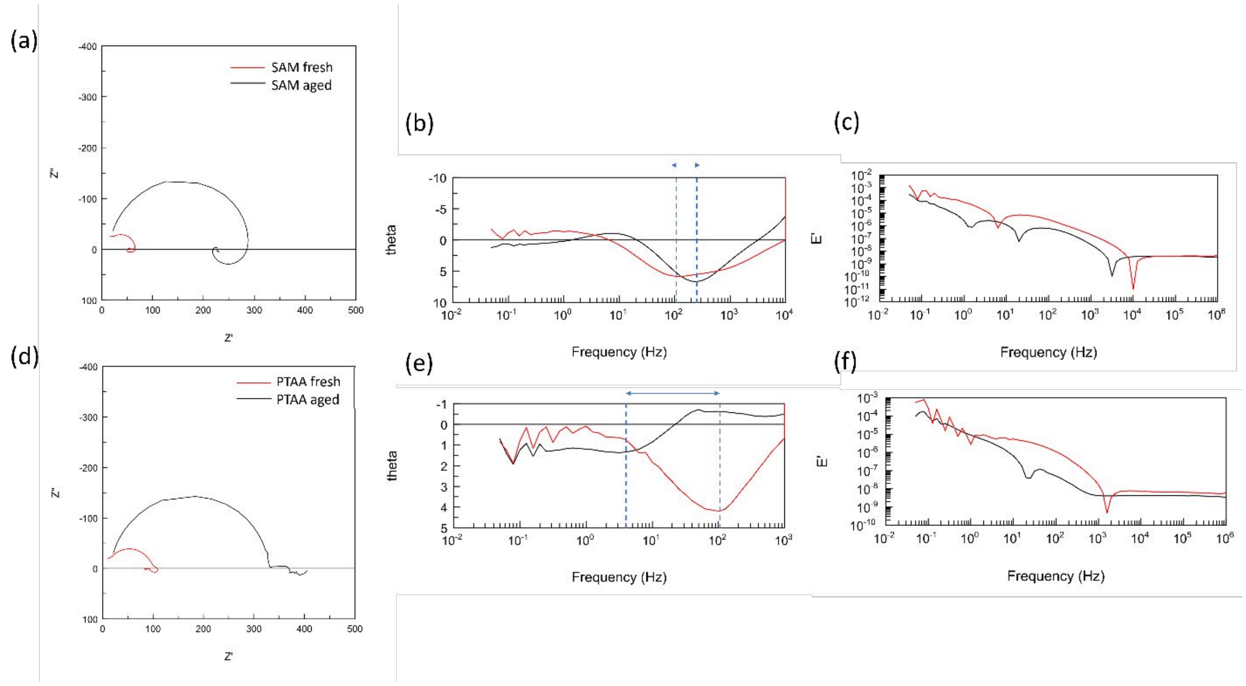

Figure S4. *Comparison of the impedance responses of fresh and aged devices. Panels (a), (b), and (c) correspond to the Nyquist plot, phase, and capacitance of SAM devices, respectively. Panels (d), (e), and (f) represent the same parameters for PTAA devices.*
Colour code: red for fresh devices, black for aged devices. Notably, for PTAA samples, ageing results in a significant shift of the time constant to much slower frequencies (e), in contrast to SAM devices, where the time constant remains nearly unchanged (b).

2. The drift-diffusion simulation do not support the mechanism proposed in Figure 5. In fact, they do not show more band bending (they show less) with increasing number of ions; they don't show more ions in the interface either; and they show more recombination with fewer ions. Additionally, the simulations increase the number of ions generally, and not the accumulation in one interface, which is not the mechanism that the authors claim. Therefore, this simulations would not support the hypothesis either. This referee is aware of the difficulty of simulating such a phenomenon, but the present simulations do not support the mechanism the authors claim.

We thank the reviewer for her/his pertinent remarks. We regret that there has been a partial misinterpretation of the data, possibly due to our very short description of the text. Figure S6 does show in fact less band bending at the PTAA due to a stronger screening of the electric field on the *perovskite side*, because of the larger concentration of ions (cations) and subsequent accumulation at the interface (Figure S4). This mechanism has been widely described in the literature (see for instance: 10.1103/PhysRevApplied.19.014061; 10.1039/D0NR03058A) and it is in line with the interpretation shown in Figure 5. On the other hand, Figure S8 reveals that the recombination rate drops in the vicinity of the PTAA surface (by a factor of approx. 6 for the lowest ion density studied). This drop becomes less pronounced when the concentration of ions is increased, approaching the bulk value at shorter distances to the surface. This is precisely the mechanism Figure 5 refers to, which describes the *interface.*

It is true, however, that the overall recombination rate across the whole active layer slightly decreases as more ions are added to the system. This is most likely due to the fact convincingly raised by the reviewer, that we are "simulating" the accumulation of ions at the interface by setting the *overall* or *bulk* density of ions. Unfortunately, the drift-diffusion model cannot modify locally the density of ions. However, we believe that the processes occurring at the interface are still correctly considered and that this is the reason why the general trends in the impedance spectra are reproduced when PTAA is replaced by SAM. The simulation also unequivocally shows that fewer ions at the interface lead to less recombination.

Changes introduced: we have modified the text as indicated:

To corroborate this qualitatively, we have performed drift-diffusion (DD) numerical simulations of the PTAA/PS/C60 structure and varying ionic densities to replicate the effect of replacing the PTAA with a chemical component, this is the SAM, which effectively reduces the number of mobile ions at the interface. Simulations were carried out with SETFOS software from Fluxim (see details in the SI). It has to be noted that in the DD model, it is not possible to modify the density of ions locally. However, by tuning the overall density of mobile ions in the active layer we are able to reproduce the change in the dark impedance spectrum and the diminution of the electron-recombination rate at the interface.

Among other errors, the authors say that the hysteresis decreases with increasing scan rates because the HI is decreasing, but it is an increase of inverted hysteresis.

We would like to clarify that the decrease in the hysteresis index evolves, inevitably, to an inverted hysteresis (negative values). The authors have previously reported a complete analysis of this behaviour (https://doi.org/10.1021/acs.jpclett.0c02331). The text has been corrected accordingly.

In light of these concerns, I believe the manuscript requires significant revision to adequately support its claims.

Additional Questions:

Urgency: Moderate
Significance: Moderate
Novelty: High
Scholarly Presentation: Moderate

Is the paper likely to interest a substantial number of physical chemists, not just specialists working in the authors' area of research? Yes

jz-2024-031946.R2

Name: Peer Review Information for "Insights from Impedance Spectroscopy in Perovskite Solar Cells with Self-Assembled Monolayers: Decoding SAM´s Tricks"

Second Round of Reviewer Comments

Reviewer: 3

Comments to the Author

Despite the detailed questions I provided, the authors' explanations lack the necessary clarity and rigor. Specifically, the Bode plots added by the authors indicate that the inductive arc occurs at lower frequencies, which would imply a higher value for τ_k. However, τ_k depends on both a fitted inductor and resistance, and I cannot discern a satisfactory fitting of any arc—indeed, there appears to be no arc present, only a concatenation of noisy data points.

Furthermore, as previously noted, the local simulation of ion accumulation due to OH reduction is not feasible with drift-diffusion (DD) simulations. While the recombination appears to decrease slightly in a very narrow region of the simulation, the mechanism remains unresolved. In fact, the surface recombination at this part of the device is so much lower than in other regions that it is implausible to consider it as having a meaningful impact on the overall performance.

In addition, I have noted that the authors' responses to questions raised by another reviewer are similarly insufficient. Many critical points remain unresolved, which further undermines the overall rigor and reliability of the study.

Given the insufficient resolution of these critical issues, I am unable to recommend the publication of this manuscript in its current form. While I appreciate the authors' efforts, substantial improvements are required for it to meet the standards of The Journal of Physical Chemistry Letters.

Reviewer: 1

Comments to the Author

The authors have made a great effort to improve the paper based on the reviewers' comments.

However, there is one critical point that still concerns me about the manuscript. The authors focus their analysis on Impedance Spectroscopy and, in particular, on the surface polarization model and its corresponding linearization that gives rise to the equivalent circuit shown in the Supporting Information. This model is useful for perovskite devices in which a coupling in the slow variable is found, i.e., the time constant of the RC branch is equal to that of the RL branch. Otherwise, the parameter b is introduced ad hoc to modify the time constants, but it has no clear physical meaning and does not come from the original nonlinear equations of the model itself. With this, I urge the authors to review the literature beyond 2017 and use improved versions of the surface polarization model that explain the experimental situation given in this paper.

Author's Response to Peer Review Comments:

Reviewer(s)' Comments to Author:

Reviewer: 3

Recommendation: Reconsider as an article in The Journal of Physical Chemistry A/B/C.

Comments:
Despite the detailed questions I provided, the authors' explanations lack the necessary clarity and rigor. Specifically, the Bode plots added by the authors indicate that the inductive arc occurs at lower frequencies, which would imply a higher value for τ□. However, τ□ depends on both a fitted inductor and resistance, and I cannot discern a satisfactory fitting of any arc—indeed, there appears to be no arc present, only a concatenation of noisy data points.

We understand the reviewer's concern regarding the actual significance of the τ□ constant. To further clarify this issue, we provide with this revision an additional analysis based on the direct extraction of the low-frequency time constants from the spectra (vertical dashed lines), with no fitting involved (results shown in Figure S4 panel (g)), where the same behaviour between aged and fresh sample is observed. In addition, in panels (c) and (f), the peak shifting to negative capacitance values can be noticeably observed. A negative capacitance (see https://doi.org/10.1021/acs.jpclett.0c02331) is a clear indication of inductive behaviour. Besides, no noisy data affects our capacitance data in the 10^0-10^2 Hz interval. We strongly believe that these observations, which do not depend on a fitting to an RL element or noisy data points, indicate that the inductive behaviour appears at very similar frequencies for SAM samples, regardless of ageing processes. However, in the case of PTAA, these frequencies shift to lower values as the sample ages.

Changes introduced: we added an extra figure S4 and corresponding discussion in the main text

Furthermore, as previously noted, the local simulation of ion accumulation due to OH reduction is not feasible with drift-diffusion (DD) simulations.

The reviewer is totally right that DD simulations cannot model from a fundamental perspective the mechanistic effect we describe in our manuscript. Reproducing local ion immobilization by terminal OH groups would require an atomistic approach, such as DFT or Molecular Dynamics, which lies beyond the scope of this manuscript. However, we still believe that DD simulations can provide valuable insights, especially because they can produce a simulated impedance spectrum as well as density and recombination profiles. These are pieces of information that cannot be obtained from an atomistic approach as it seems to be suggested by the reviewers. Using DD simulations, we can analyse the impact of an overall modification of the ion density. The calculations show (Figure S5) that more ions lead to inductive behaviour, and that this effect is more pronounced the more ions are present, the same behaviour is observed when the SAM and PTAA samples are compared. The low-frequency signals appear in the same intervals as in the experiments (10^0-10^2 Hz interval), which is a remarkable result considering that we used realistic concentrations of ions in perovskites. Knowing that ions have their primary impact on the interface, where they accumulate (see refs 3, 19, 21, 30), we still believe that this piece of information is useful and insightful and deserves to be included in the discussion of our results.

While the recombination appears to decrease slightly in a very narrow region of the simulation, the mechanism remains unresolved. In fact, the surface recombination at this part of the device is so much lower than in other regions that it is implausible to consider it as having a meaningful impact on the overall performance.

The reviewer's concern is pertinent as it points to the fact that the overall recombination loss in a perovskite solar cell can take place mainly in the bulk. In this respect, we would like to highlight that the main aspect of this work is to explain why SAM samples are not only more stable than the PTAA ones but also present higher values of V_{oc}. Thus, we focus on the impact of replacing PTAA with SAM, and this replacement affects the HTL/perovskite interface only. In this respect, the DD simulations show that fewer ions at the interface are linked with a reduction of the recombination rate at the interface (DD simulations do allow us to look at *local* effects in the recombination rate).

Extrapolating this observation to the interpretation of the experiments, it is important to note that interfacial recombination is a key source of photovoltage loss and that this is particularly relevant for high-performance devices where bulk recombination is minimized, making surface recombination a dominant loss mechanism. In addition, mobile ions are known to have deleterious effects on perovskite solar cell's performance. For instance, Thiesbrummel et al. (10.1002/aenm.202101447) showed using DD simulations in a p-i-n configuration, very similar to ours, that accumulation of ions at the interface inhibits charge extraction, hence triggering more recombination. Lammar et al. (10.1039/D2TA04840J) also demonstrated by comparing DD calculations and experimental samples with deliberate formation of perovskite defects, that more ions lead to photocurrent and photovoltage losses.

In addition, I have noted that the authors' responses to questions raised by another reviewer are similarly insufficient. Many critical points remain unresolved, which further undermines the overall rigor and reliability of the study.

We take note of the reviewer's remark and kindly refer to our response to Reviewer 1. There we also reinforce the validity of DD simulations to our case by showing in Figure S7 how the concentration of holes is 2 orders of magnitude larger than the concentration of electrons, as referred to in the work reported by Clarke et al. as the identifier of the presence of inverted hysteresis.

Reviewer: 1

Recommendation: This paper may be publishable, but major revision is needed; I would like to be invited to review any future revision.

Comments:
The authors have made a great effort to improve the paper based on the reviewers' comments.

However, there is one critical point that still concerns me about the manuscript. The authors focus their analysis on Impedance Spectroscopy and, in particular, on the surface polarization model and its corresponding linearization that gives rise to the equivalent circuit shown in the Supporting Information. This model is useful for perovskite devices in which a coupling in the slow variable is found, i.e., the time constant of the RC branch is equal to that of the RL branch. Otherwise, the parameter b is introduced ad hoc to modify the time constants, but it has no clear physical meaning and does not come from the original nonlinear equations of the model itself. With this, I urge the authors to review the literature beyond 2017 and use improved versions of the surface polarization model that explain the experimental situation given in this paper.

We sincerely thank the referee for their insightful comments and for emphasizing this important point. Indeed, more advanced versions of the Surface Polarization Model (SPM) have been reported recently. The Modified Surface Polarization Model (m-SPM), as presented by Clarke et al.

(https://doi.org/10.1063/5.0136683) and further elaborated in a recent didactic study (https://doi.org/10.1002/aenm.202400955), offers a higher degree of physical precision than the version used in our manuscript. By incorporating the role of large hole populations in the perovskite bulk and adopting a more realistic non-linear potential distribution, the m-SPM provides an improved framework for predicting hysteresis in PSCs. In this respect, it is important to note that we also observe larger concentrations of holes (by 2 orders of magnitude) with respect to that of electrons in our DD simulations (Figure S7). This explains the occurrence of inverted hysteresis in line with the predictions of the m-SPM.

Having said that, we fully acknowledge the significance of these contributions to the field and agree on the importance of citing these works appropriately. However, we believe that the use of the original SPM in our study does not alter our final conclusions, which remain well-supported by the drift-diffusion (DD) simulations we have presented. As indicated in our response to reviewer 3 (see above), the trend of τ_{kin} obtained from the fitting using the equivalent circuit (EC) from the standard SPM aligns perfectly with the time constant directly extracted from the impedance spectra (Cole-Cole plot), as demonstrated in the figure below. This agreement corroborates that the processes in PTAA-based devices are significantly slower than those in SAM-based devices, highlighting the stronger influence of ionic motion in the former and its much lesser impact in the latter.

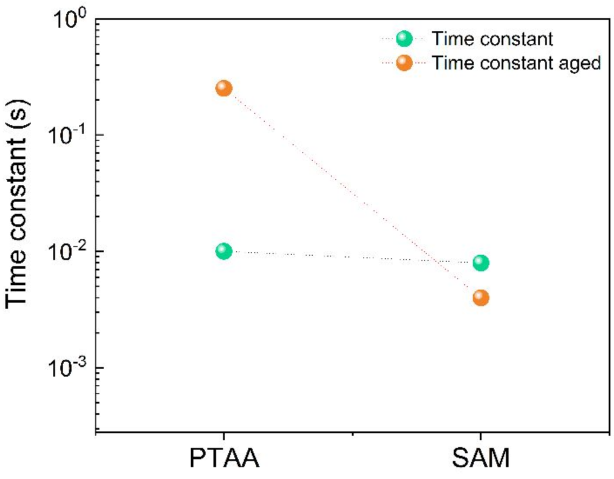

Changes introduced: "A recent work reported by Clarke et al. analyses these effects in depth, predicting the presence of inverted hysteresis by introducing a modified surface polarization model (m-SPM).22 that simplifies and helps to interpret the DD simulations."

We have included this figure in the SI with an extended explanation in this regard:

"(…) This phenomenon has been previously modelled by adding an R_L-L branch to the equivalent circuit typically used to fit the impedance spectra. The time constant associated with this R_L-L branch, denoted as τ_{kin}, increases when the inductive behaviour becomes more pronounced.24,25(see Supporting Information for details). The model used here associated with the equivalent circuit to fit the impedance response is related to the surface polarization model (SPM), widely reported and discussed in the literature. The SPM model was introduced to reduce the complexity of the DD analysis and facilitate the understanding of hysteresis. A more accurate version of this model was recently developed by Richardson and co-workers. (REF) This modified surface polarization model (m-SPM) was introduced to provide a rationale for the

observation of inverted hysteresis, as is the case here. The m-SPM keeps the main approximation of the original SPM (that the concentration of ions is much larger than that of electronic carriers – see Figure S7) but introduces the additional consideration that holes outnumber electrons due to the extraction barriers at the contacts. We note that this is also what we observe in the simulations we run (Figure S7) and that reproduce quite accurately the experiments."

"(…) Considering this, we analyzed the impedance spectra of PTAA and SAM samples using the equivalent circuit that includes the inductive behaviour (R_L-L branch). We would like to remark that the controversy presented in the field regarding the use of different models with different grades of accuracies and, foremost, different equivalent circuits to interpret the physical processes governing perovskite devices, is well known and much less negligible. This is the reason why many authors decide to focus just on the time constants directly extracted from the impedance spectra (Cole-cole plot) without the need for a fitting. We would like to show here that the use of the standard SPM and its corresponding equivalent circuit associated, provides a trend of τ_{kin} values that fully agrees with the time constants directly extracted from the impedance plot, being well supported as well by the drift-diffusion simulations (DD) we have presented (available in the SI). In the following, we will discuss the values obtained both from the fitting using the EC and the direct extraction from the Cole-Cole plot of the impedance response.

Fitting results are presented in Table 1 together with the time constants extracted from the impedance plot."
